# Non-dikarya fungi share the TORC1 pathway with animals, not with *Saccharomyces cerevisiae*

**DOI:** 10.1038/s41598-025-89635-4

**Published:** 2025-02-18

**Authors:** Drishtee Barua, Magdalena Płecha, Anna Muszewska

**Affiliations:** Institute of Biochemistry and Biophysics, Polish Academy of Sciences, Pawińskiego 5A, Warsaw, 02-106 Poland

**Keywords:** Target of rapamycin, Activation, Inhibition, Starvation, Non-dikarya, Fungal biology, Fungal evolution, Fungal genomics, Protein sequence analyses, Sequence annotation, Nutrient signalling

## Abstract

Target of rapamycin (TOR), discovered in *Saccharomyces cerevisiae*, is a highly conserved serine/threonine kinase acting as a regulatory hub between the cell and its environment. Like mammals, in fungi, the TOR complex 1 (TORC1) pathway is essential for coordinating cell growth in response to nutrient availability. The activation of TORC1 is similar in yeast and mammals, while its inhibition is more complex in mammals. This divergence of TORC1 regulation opens the question of how common are the yeast and mammalian variants in the fungal kingdom. In this work, we trace the evolutionary history of TORC1 components throughout the fungal kingdom. Our findings show that these fungi contain the mammalian-specific KICSTOR complex for TORC1 inhibition. They also possess orthologs of serine, arginine and methionine sensors of TORC1 pathway that orchestrate the response to nutrient starvation in mammals. The Rheb-TSC mediated activation of mammalian TORC1 that was lost in *Saccharomycotina* was also conserved in non-Dikarya. These findings indicate that the TORC1 pathway in non-Dikarya fungi resembles mammalian TORC1. *Saccharomycotina* lost many of the inhibitory components and evolved alternate regulatory mechanisms. Furthermore, our work highlights the limitations of using *S. cerevisiae* as a fungal model while putting forward other fungi as possible research models.

**Supplementary Information:**

The online version contains supplementary material available at 10.1038/s41598-025-89635-4.

## Introduction

Target of rapamycin (TOR) protein is a member of the phosphoinositide-3-kinase related protein kinase family. It was first discovered in *Saccharomyces cerevisiae* through genetic screening of mutants resistant to the inhibitory effects of rapamycin^1^. Later on, it was also documented in filamentous fungi and described in other non-pathogenic fungi^2^ and in animals^3^. TOR signaling pathway is conserved in diverse eukaryotes, from yeast to humans and serves as a central regulatory hub for nutrient-sensing and maintaining cellular homeostasis^4^. In the presence of nutrients, the TOR pathway promotes cell growth by stimulating anabolic processes such as transcription, translation and ribosome biogenesis, while repressing catabolic processes such as autophagy^5^. Here, we introduce two best-known TORC1 pathway versions: the *Saccharomyces cerevisiae* (referred to as yeast) and *Homo sapiens*. For convenience, we introduce the gene names of both systems in Table 1.

### mTOR

Mammals possess one TOR protein kinase, mTOR, which acts as a catalytic subunit for two complexes: mTOR complex 1 (mTORC1) and mTOR complex 2 (mTORC2). The former regulates cell growth while the latter is responsive to insulin-like growth factor 1 (IGF-1) signaling^3^. mTORC1 and mTORC2 share regulatory subunits mLST8 and Deptor. Both mTOR complexes have specific substrate recognition and regulatory components (PRAS40 for mTORC1 and mSin1 for mTORC2).

### ScTOR

Budding yeasts on the other hand, possess two TOR proteins, Tor1 and Tor2^1^ each of them forming a distinct complex, TORC1 and TORC2 respectively. Both complexes include a common regulatory protein Lst8 that functions as their primary interactor. TORC1 includes a novel component found in yeast, Tco89, which maintains cellular integrity during rapamycin treatment and stress^6^. Similarly, TORC2 has a yeast-specific component Avo2 that downregulates TOR signaling^7^.

**Table 1:** List of TORC1 subcomplexes and pathway proteins.

Sub complex	Subunit	mTOR (*Homo sapiens*)	ScTOR (*Saccharomyces cerevisiae*)	Filamentous Dikarya (*Magnaporthe oryzae*, *Blumeria graminis tritici*)	Pfam/Interpro accession	Function/interactions
TORC1	TOR1	mTOR1	Tor1	Tor1	PF00454 PF02259 PF02260 PF08771	Central controller of cell growth^8^
	Lst8	mLst8	Lst8	Lst8	PF00400	Binds to kinase domain of TOR^3^
	Raptor	Rptor	Kog1	Kog1	PF14538	Acts as an adaptor to recruit substrates to mTOR^3^
	Deptor	Dptor			PF00610	Binds directly to mTOR to reduce its activity^3^
	PRAS40	PRAS40			PF15798	Binds to both Raptor and TOR to inhibit mTORC1 activity^3^
FLCN-FNIP1/Lst7-Lst4	FLCN	FLCN/Bhd1	Lst7	Lst7	PF11704 PF16692	Interacts directly with RagA/RagC^3^
	FNIP1	FNIP1	Lst4	Lst4	PF14636 PF14637 PF14638	Interacts directly with RagA/RagC^3^
RAGULATOR/LAMTOR/EGO	Lamtor1	Ltor1	Ego1/Meh1		PF15454	Binds with C-terminal roadblock domain of RagA/RagC or Gtr1/Gtr2^3^
	Lamtor2	Ltor2			PF03259	Binds with C-terminal roadblock domain of RagA/RagC^3^
	Lamtor3	Ltor3			PF08923	Forms roadblock domain dimer with Lamtor2 & interacts with Lamtor1–5^9^
	Lamtor4	Ltor4			IPR034601	Forms incomplete roadblock domain dimer with Lamtor5^9^
	Lamtor5	Ltor5			PF16672	Forms incomplete roadblock domain dimer with Lamtor4^9^
	Ego2		Ego2		PF11503	Held together by Ego1 to interact with Gtr1/Gtr2^9^
	Ego3		Slm4		PF16818	Binds with C-terminal roadblock domain of Gtr1/Gtr2^3^
Rag GTPase	Rag A/B	Rag A/B	Gtr1	Gtr1	PF04670	Forms heterodimer and interacts with mTORC1 in GTP-bound state via RAGULATOR^3^
	Rag C/D	Rag C/D	Gtr2	Gtr2	PF04670	Forms heterodimer and interact with mTORC1 in GDP-bound state via RAGULATOR^3^
N/A	Rheb	Rheb	Rhb1	Rheb	PF00071	Binds to mTOR directly via lysosomal surface^3^
GATOR1/SEACIT	Depdc5	Depdc5	Sea1/Iml1	Sea1/Iml1	PF19418	Central protein that binds Nprl2 & Nprl3 and RagA/RagC^3^
	Nprl2	Nprl2	Npr2	Npr2	PF06218	Interacts with Nprl3 and associates with DEPDCC5^3^
	Nprl3	Nprl3	Npr3	Npr3	PF03666	Interacts with Nprl2 and associates with DEPDCC5^3^
GATOR2/SEACAT	Wdr24	Wdr24	Sea2	Sea2	PF00400	Catalytic subunit for ubiquitination of Nprl2^10^
	Wdr59	Wdr59	Sea3	Sea3	PF00400	Forms a dimer with Sects. 13^11^
	Mios	Mios	Sea4	Sea4	PF21719 PF21720	Interacts directly with Castor1^11^
	Seh1l	Seh1l	Seh1	Seh1	PF00400	Forms a dimer with Sea4 and localize to lysosomes^11^
	Sec3	Sec3	Sec3	Sec3	PF00400	Forms a dimer with Sea3^11^
CASTOR	Castor1	Castor1			PF18700	Forms a homodimer and binds to GATOR2 upon arginine sensing^12^
	Castor2	Castor2			PF21389	Forms a heterodimer with Castor1^12^
TSC	Tsc1	Tsc1		Tsc1	PF04388	Forms homodimer and stabilizes Tsc2 activity^3^
	Tsc2	Tsc2		Tsc2	PF03542	GAP (GTPase-activating protein) activity for Rheb^3^
KICSTOR	KICS2	KICS2/C12orf66			PF09404	Forms heterodimer with SZT2^13^
	ITFG2	ITFG2			PF15907	Forms heterodimer with KPTN^13^
	KPTN	KPTN			IPR029982	Forms heterodimer with ITFG2^13^
	SZT2	SZT2			IPR033228	Central regulator of KICSTOR that interacts with GATOR1^13^
N/A	Samtor	Samtor			PF11968	Interacts directly with GATOR1 upon methionine sensing^3^
N/A	Sestrin1	Sestrin-1			PF04636	Interacts directly with GATOR2 upon leucine sensing^3^
N/A	Tco89		Tco89		PF10452	Interacts with TOR1 similar to Lst8 and Kog1^6^

Protein names, function and PFAM accessions of protein domains are provided for each of the mammalian (mTOR) and *Saccharomyces cerevisiae* (ScTOR) components. The aliases for genes from Pezizomycotina—filamentous Ascomycota such as *Magnaporthe oryzae* and *Blumeria graminis* *tritici* are derived from Song et al. (2024).

### Upstream TORC1 signaling

**Fig. 1:**
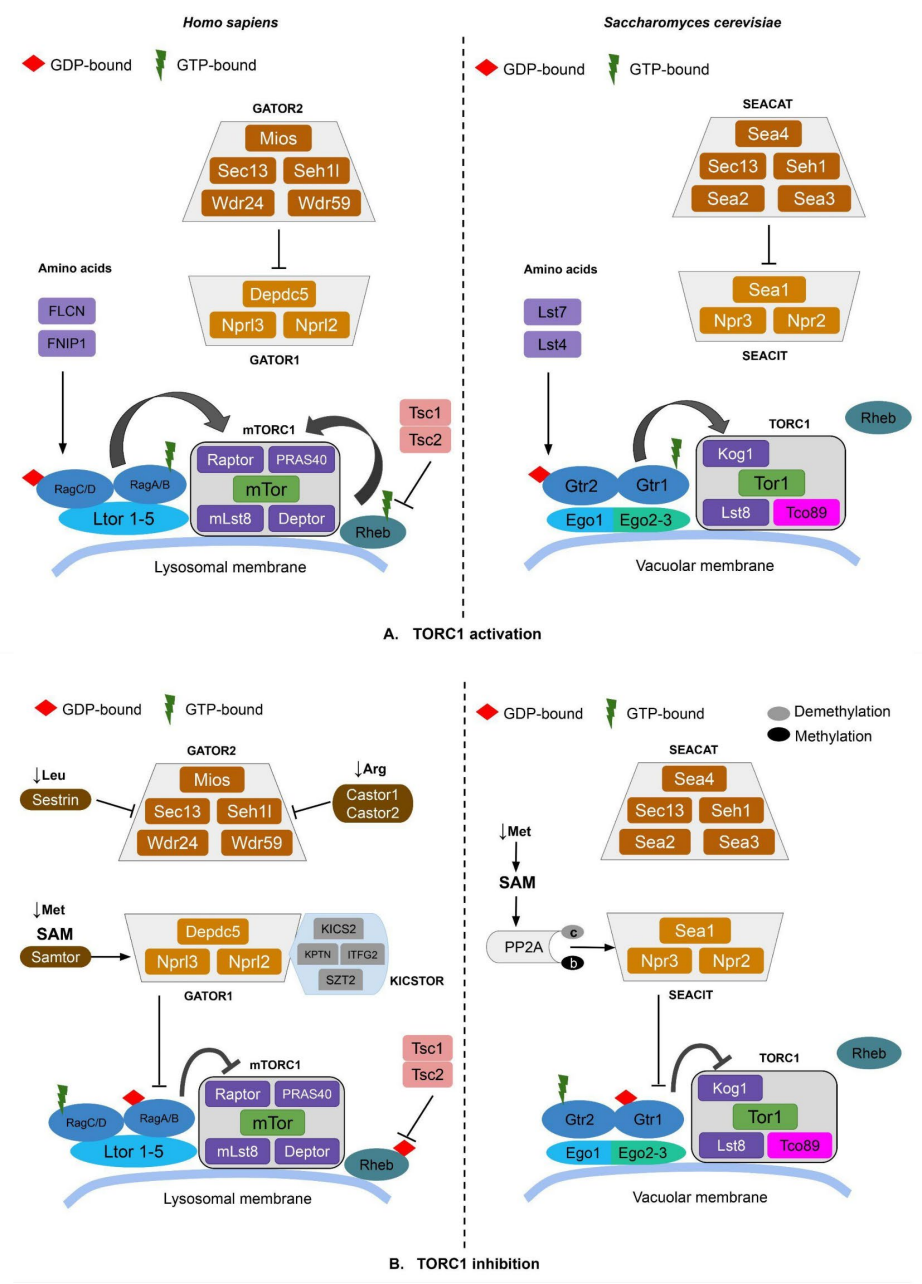
Schematic representation of the TORC1 pathway in humans and yeast, derived from Refs^3,19^. Proteins that share homology at the same position are depicted with the same color. The protein nomenclature follows Table 1.

Under nutrient-rich conditions, presence of amino-acid activates the Rag GTPases (Gtr1/2 in yeast) that interact with LAMTOR/RAGULATOR scaffolds via roadblock domains. The RAGULATOR complex then regulates their translocation from cytosol to the surface of lysosomes in mammals (and vacuoles in yeast)^14^ (Fig. 1). While mammals have five LAMTOR proteins, the RAGULATOR in *S. cerevisiae* is reduced to a three-protein EGO complex. The EGO complex similarly interacts with the Gtr1-2 GTPases as LAMTOR does with Rag GTPases^9^. Ego1 is a homolog of Ltor1, while Ego2 and Ego3 are analogs with a similar structure to human Ltor2 and Ltor3^9^.

In parallel, mTORC1 is also regulated by a GTP-bound active Rheb, which binds to mTORC1 65 Å away from the kinase domain, thereby increasing its effective substrate concentration and adopting the active conformation^15^. During nutrient starvation, the TSC complex causes hydrolysis of GTP bound to Rheb , keeping Rheb in a GDP-bound state and thereby, preventing it from activating mTORC1^16^ (Fig. 1). Yeasts, in particular *S. cerevisiae*, contain Rheb but do not have TSC orthologs and hence, lack the Rheb-dependent regulation of TORC1^3^.

### Downstream TORC1 signaling

TOR signaling pathway is also governed by the GATOR/SEA-dependent regulation that helps maintain the localization of TORC1 at the lysosomal/vacuolar membrane during nitrogen starvation (Fig. 1). It comprises GATOR1 and GATOR2 subcomplexes (SEACIT and SEACAT in yeast)^3^. GATOR2 inactivates TORC1 by binding to GATOR1 (a complex that has a GTPase-activating protein (GAP) activity towards Rag A/B) which converts the GTP-bound active Rag A/B into GDP-bound inactive state^1^. The KICSTOR complex acts as a scaffold that recruits GATOR1 to the lysosomal surface^13^. In the absence of nutrient availability, the arginine sensing protein Castor interacts with GATOR2 by forming a dimer of Castor1 and Castor2^17^. At the same time, the leucine sensor Sestrin1 binds to GATOR2. This binding induces GATOR1 activity by releasing its attachment to GATOR2^18^. The starvation of amino acid methionine involves direct sensing in mammals using Samtor, which acts as the SAM (S-adenosylmethionine) binding effector^19^. Samtor contains a class I Rossman fold methyltransferase domain that binds S-adenosylmethionine (SAM), facilitating its interaction with KICSTOR bound GATOR1 in TORC1 inhibition^20^. The tumor suppressor gene FLCN (Lst7 in yeast) forms a complex with FNIP1/2 (Lst4 in yeast), and acts as a GAP towards Rag C/D. This promotes the binding of mTORC1 to the Rag heterodimers^21^. This coordination by the FLCN/FNIP1 complex contributes to metabolic homeostasis in mammals^22^.

*S. cerevisiae* do not have Samtor, and employ an indirect methionine sensing mechanism through protein phosphatase 2 A (PP2A) methylation. Decreased methionine levels lead to demethylation of the PP2A catalytic subunit PP2Ac, resulting in an inactivated phosphatase. This in turn, dephosphorylates Npr2 (mammalian Nprl2) and activates the SEACIT (GATOR1) mediated TORC1 inhibition^19^. 

Shertz and co-workers in 2010 annotated the TORC1 pathway components in Zygomycetes (now Mucoro- and Zoopagomycota) and Chytridiomycota, a set of Dikarya members along with non-fungal Opisthokonta. They reported conservation of the Tor1 (mTor) kinase protein architecture among eukaryotes, along with duplications in most fungal groups^2^. Upstream regulators Tsc1 and Tsc2 comprising the TSC complex were identified in most fungal lineages except *S. cerevisiae*. A study by Manning and Cantley pointed out that loss of function of Rheb resulted in growth arrest phenotype in *Schizosaccharomyces pombe*^23^. A recent study on conservation of TORC1 nutrient signaling pathway in arbuscular mycorrhizal (AM) fungi pointed to the absence of yeast proteins Ego1, Ego3 and Tco89 in AM fungi^24^. This opened the question of how universal the *Saccharomyces cerevisiae* TORC1 pathway is to the fungal tree of life.

To address this question, we used mammalian and *S. cerevisiae* TORC1 pathway models as reference. We report the presence of different amino acid-specific sensor proteins, animal-specific regulatory complexes typical of mTOR across non-Dikarya fungi. Our findings suggest a mammalian mode of TORC1 pathway architecture in non-Dikarya fungal lineages. The possible implications of having an mTORC1-like mode of regulation starts from nutrient-sensing specificity in the environment, the balance of cell energetics via possible growth limitation strategies to pathogenicity regulation along with metabolite production.

## Results

### Distribution of TORC1 pathway components

**Fig. 2:**
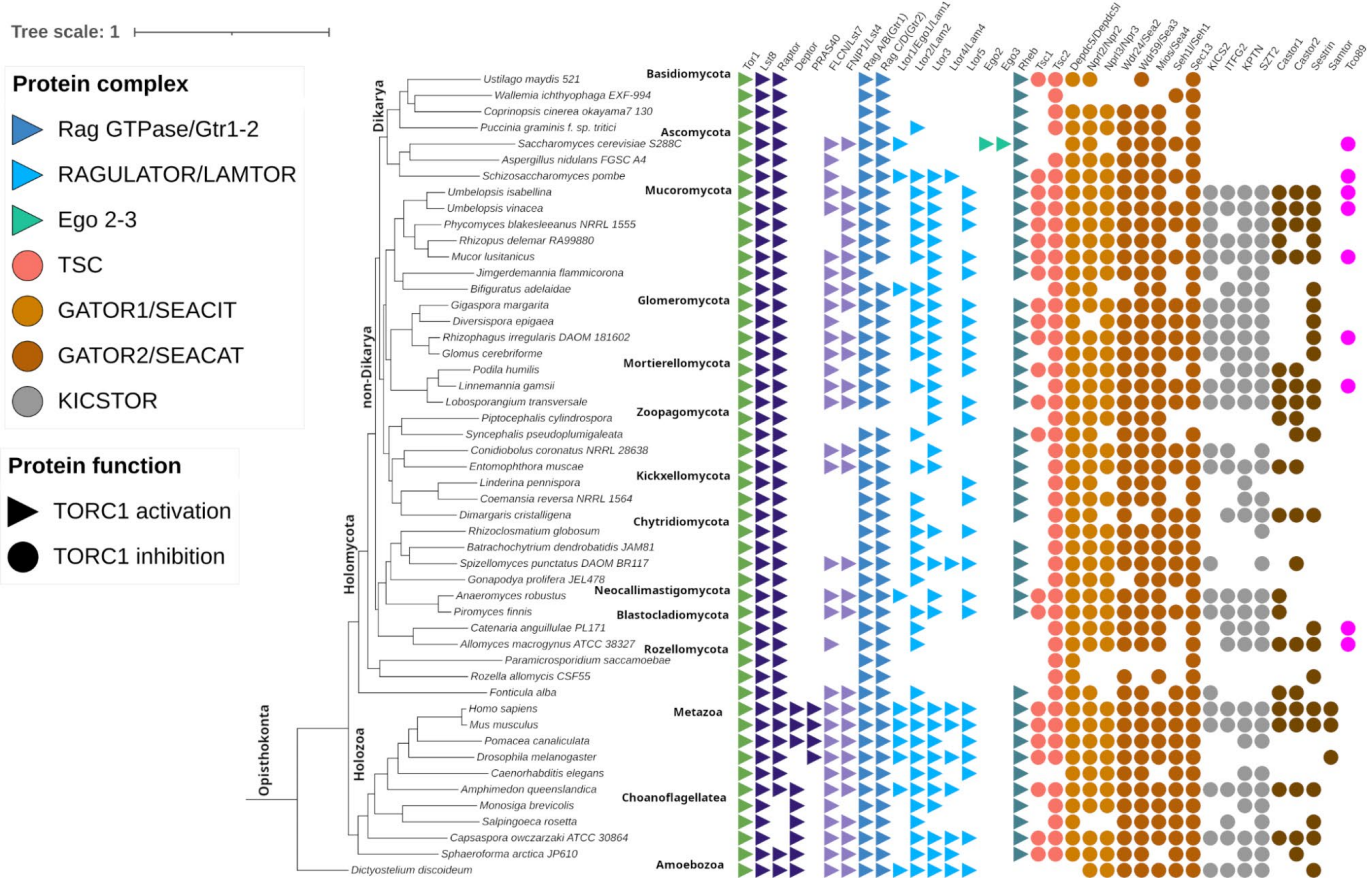
Distribution of 36 TORC1 pathway components among model eukaryotes with at least two representatives from each fungal lineage. The nomenclature of proteins is in accordance with TORC1 protein names in *Homo sapiens* as listed in Table 1. The phylogenomic tree was built in Orthofinder and drawn in iTOL. The taxonomic ranks are in accordance with Voigt and coworkers ^25^.

As the constituents of TORC1 regulation differ between mammals and yeasts, we looked for their distribution across the fungal kingdom, along with Metazoa and early Opisthokonts as outgroups. 32 out of 36 mammalian TORC1 components were identified in fungal proteomes (Fig. 2). It was interesting to find components of the human RAGULATOR complex (Ltor1-5) distributed not only across Early Diverging Fungi (EDF) but also in a few Dikarya members (Fig. 2). The homologs of Ltor2 found in Ascomycota were truncated, containing an N-terminal roadblock domain of 60 amino acids (Fig. 2).

The GATOR/SEA complex (combination of SEACIT and SEACAT complexes) of TORC1 inhibition pathway showed conservation across eukaryotes. Homologs of the mammalian KICSTOR, TSC complex and Sestrin were conserved across the phyla Mucoromycota (Mucoro-, Mortierello- and Glomeromycota), Entomophthoro- and Neocallimastigomycota, and scattered in the remaining EDF (Fig. 2). Loss of Castor and FLCN-FNIP1 proteins were observed in Rozello-, Chytridio- and Zoopago-, Kickexello- and Glomeromycota. It is worth noting that Dikarya members Ustilago- and Taphrinomycota possessed a complete TSC complex along with Rheb, pointing to the possible function of Rheb-dependent TORC1 activation (Fig. 2). The yeast-specific Tco89 protein was found in Mucoro- and Blastocladiomycota, along with duplications in Mucoromycota (Supplementary File S1).

The LAMTOR equivalent EGO complex is known only in *S. cerevisiae*. In order to assess its occurrence in other Saccharomycotina members, we based our search on proteomes from the y1000 + project^26^ encompassing a total of 1154 Saccharomycotina species.

The analysis of EGO complex proteins from 1154 Saccharomycotina members identified 575, 165 and 317 homologs for Ego1, Ego2 and Ego3, respectively. All 3 EGO complex subunits were present only in 6 out of 17 families (Saccharomycetaceae, Debaryomycetaceae, Saccharomycodaceae, Dipodascaceae, Wickerhaomycetaceae and Phaffomycetaceae). We observed that the oldest lineages of Saccharomycotina retained both EGO and LAMTOR components with a co-existence of both almost complete complexes in Trichomonascaceae. Two families Sporopachydermiaceae and Saccharomycopsidaceae have neither EGO nor LAMTOR components. This observation suggests a gradual exchange of the LAMTOR with EGO across the Saccharomycotina families and common losses of EGO components in Saccharomycotina (Supplementary file S1).

### Transcriptomics of TORC1 pathway proteins

**Fig. 3:**
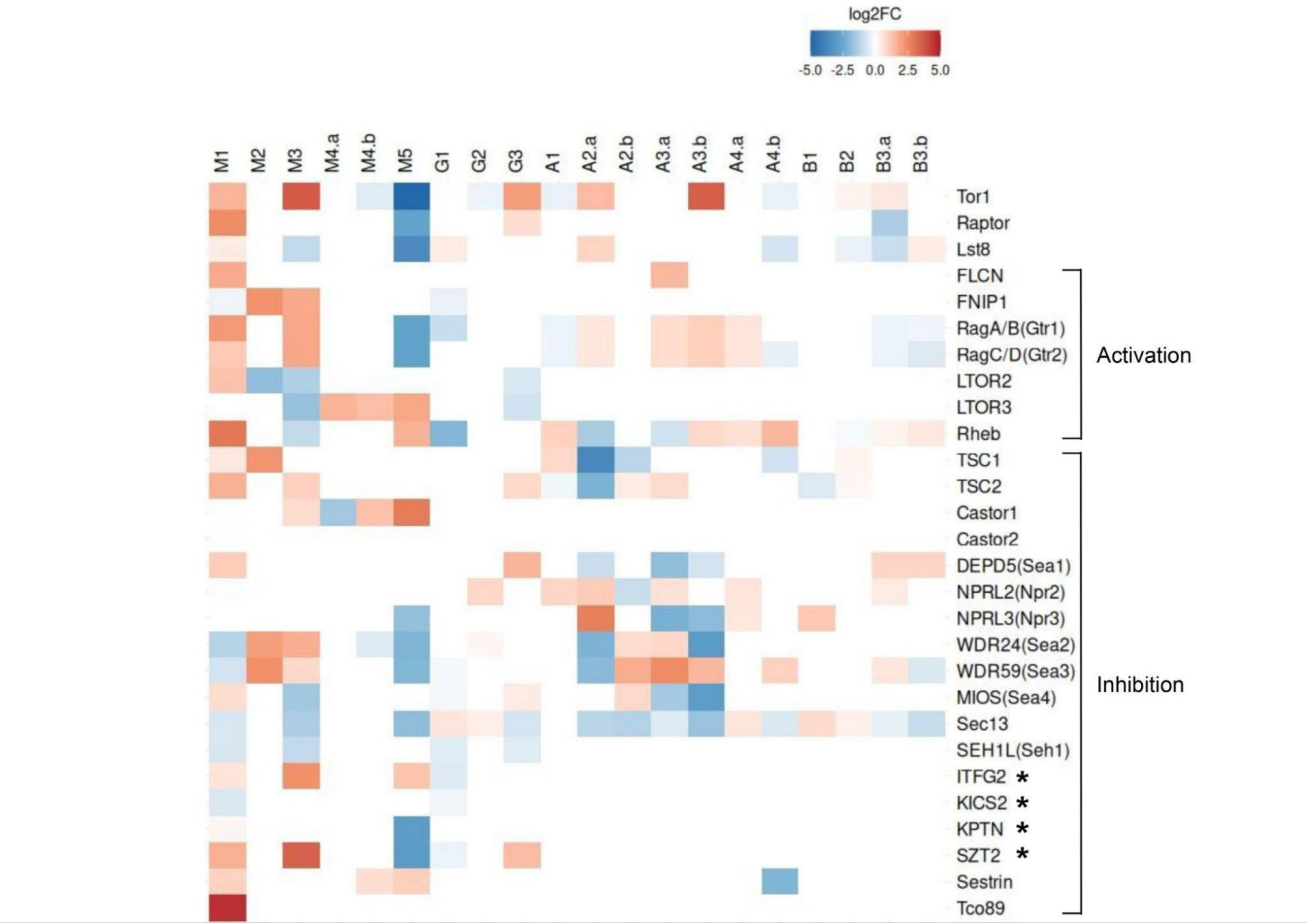
Differential expression profiling of 28 TORC1 pathway genes expressed in different environmental conditions in members of Mucoromycota **(M)**, Glomeromycota **(G)**, Ascomycota **(A)** and Basidiomycota **(B)**. The empty cells signify the absence of gene expression from the organism in the given environmental condition. Note that columns **A1–B3.b** lack the homologs of genes marked with *. **M1**—*Mucor lusitanicus* growth in anaerobic vs. aerobic conditions; **M2**—*Rhizopus microsporus* growth in presence vs. absence of murine macrophage; **M3**—*Rhizopus delemar* growth in presence vs. absence of murine macrophage; **M4(a)**—*Rhizopus delemar* host-pathogen interaction (6 h) using human airway epithelial cells (A549); **M4(b)**—*Rhizopus delemar* host-pathogen interaction (16 h) using human airway epithelial cells (A549); **M5**—*Rhizopus delemar* host-pathogen interaction (18 h) using mouse bone marrow-derived macrophages (BM); **G1**—*Gigaspora rosea* response to plant signals in the switch from asymbiotic to presymbiotic growth; **G2**—*Rhizophagus irregularis* growth in *Lotus japonicus* roots upon exposure to high vs. low concentrations of phosphate (500µM, 300 µM vs. 100 µM, 20 µM); **G3**—*Rhizophagus irregularis* association with *Medicago truncatula* and treatment with strigolactone for 24 h; **A1**—*Trichoderma reesei* QM6a and Δace1 strains grown in Mandels-Andreotti medium containing 1% D-mannitol as carbon source; **A2(a)**—*Neurospora crassa* wild-type strain treated with 20mM H2O2; **A2(b)**—*Neurospora crassa* upf1 knockout strain treated with 20mM H2O2; **A3(a)**—*Coccidioides immitis* gene expression profiling in young spherules; **A3(b)**—*Coccidioides immitis* gene expression profiling in mature spherules; **A4(a)**—*Orbilia oligospora* wild-type gene expression profiling of cAMP/PKA signaling pathway; **A4(b)**—*Orbilia oligospora* ΔAoPkaC1 gene expression profiling of cAMP/PKA signaling pathway; **B1**—*Coprinopsis cinerea* gene expression profiling upon deletion of *cre1* regulating carbon catabolite repression; **B2**—*Rhizoctonia solani* AG1-IA transcriptome profiling upon infecting rice leaves; **B3(a)**—*Ustilago maydis* wild-type gene expression profiling in minimal medium (2% arabinose/glucose) after 24 h of growth; **B3(b)**—*Ustilago maydis* Pcrg1:grx4 strain gene expression profiling in minimal medium (2% arabinose/glucose) after 24 h of growth.

The presence of mRNA in transcriptomic data provides evidence of the gene being functional and active in a given organism. To examine the functionality of the candidate TORC1 genes in EDF, we analyzed the expression profiles of available transcriptomic data in EDF from various environmental conditions. A total of 28 out of 36 predicted genes encoding TORC1 pathway components were expressed (Fig. 3). For instance, the transition from aerobic vs. anaerobic growth of *M. lusitanicus*^27^ (Fig. 3, **M1**) changed the expression of 22 out of 28 predicted TORC1 pathway genes. The upregulation of genes was observed for a few of the TORC1 activation (FLCN, Rag GTPases, Ltor2, Raptor, Sin1, Rheb) and inactivation (GATOR2 complex, Sestrin, Tco89, and the KICSTOR complex protein SZT2) components, while GATOR1 complex genes *DEPDC5*, *NPRL2* and *NPRL3* were downregulated. Genes for KICSTOR complex proteins were both upregulated (*SZT2*) and downregulated (*KICS2*). In another study, *R. microsporus* exposed to murine macrophages^28^ (Fig. 3, **M2**) showed upregulation of Rag GTPases, Tsc1 and KICSTOR components ITFG2 and SZT2, and downregulation of Rheb and RAGULATOR components Ltor2 and Ltor3, while a similar study on *R. delemar* (Fig. 3, **M3**) found only Tsc1 upregulated. A dataset on *R. delemar* infection on A549 human airway epithelial cells^29^ for 6 h (Fig. 3, **M4(a)**) and 16 h (Fig. 3, **M4(b)**) showed downregulation of Castor1 in the former and upregulation in the latter stage of infection. The gene encoding Tor1 kinase protein was also downregulated in the 16 h infection phase, pointing to the inhibition of TORC1 pathway as a response to infection. In another dataset of mouse bone-marrow derived macrophages (BMDMs) infected with *R. delemar* for 1 h, 4 h and 18 h^30^, we found the expression of TORC1 pathway genes from samples infected for 18 h (Fig. 3, **M5**). Both TOR kinase and Rag GTPases were downregulated, along with KICSTOR complex proteins KPTN and SZT2, and GATOR components Nprl3 (Npr2) and Wdr24 (Sea2). Castor1 and Sestrin along with Rheb were upregulated. The study involving *G. rosea* treatment with G24, a synthetic analog of strigolactone, in its response to plant signals during the switch from asymbiotic to presymbiotic growth^31^ (Fig. 3, **G1**), observed a downregulation of 6 out of 8 (Tor1, FNIP1, Rag A/B, Rheb, Seh1, ITFG2) TORC1 components. The gene expression profiling of *R. irregularis* roots exposed to higher phosphate concentrations (300 µM, 500 µM)^32^ (Fig. 3, **G2**) identified a downregulated Tor1 kinase and an upregulated Nprl2. Another study on *R. irregularis* focused on differential expression of strigolactone-treated spores a day after inoculation^33^ (Fig. 3, **G3**) identified an upregulated Tor1, Raptor, Tsc2, Depdc5, SZT2, and downregulated RAGULATOR (Ltor2, Ltor3) and GATOR (Sect. 13, Seh1/Seh1l) proteins.

We also looked at the transcriptomic profiles of experiments obtained from Dikarya members. We observed a downregulation of Rag GTPases alongside upregulation of Rheb, Tsc1 and Nprl2 in *T. reesei* strains grown in carbon-deficit source (1% D-mannitol) (Fig. 3, **A1**). In another study of stress response against oxidative stress (treatment with 20mM H_2_O_2_) in *N. crassa*^34^ wild-type and upf1 knockout strains, TORC1 complex, Rag GTPases and GATOR1 components were upregulated in wild-type strains (Fig. 3, **A2(a)**). The upf1 knockout strains exposed to H_2_O_2_ showed an upregulated GATOR2 and a downregulated TSC complex (Fig. 3, **A2(b)**). The expression profiling of *C. immitis*^35^ in young and mature spherules displayed an upregulated Tor1 along with its activation components in mature spherules (Fig. 3, **A3(b)**) and downregulation of its inhibitory counterparts. The young spherules however, displayed low signals of upregulation of TORC1 pathway activation and higher signals in its downregulation counterparts (Fig. 3, **A3(a)**). Expression profiling of the cAMP/PKA signaling pathway in *O. oligospora*^36^ wild-type (Fig. 3, **A4(a)**) and ΔAoPkaC1 (Fig. 3, **A4(b)**) strains displayed low signals of upregulation of TORC1 pathway components in the former and downregulation in the latter.

Another study involving gene expression profiling of the basidiomycete *C. cinerea* upon deletion of cre1 gene (regulating carbon catabolite repression)^37^ (Fig. 3, **B1**) displayed upregulation of Tor1 and GATOR1 (Nprl3, Sect. 13) proteins, along with a downregulation of Tsc2. The *R. solani* AG1-IA strain upon infection of rice leaves (Fig. 3, **B2**) showed only a downregulated Lst8. The gene expression profiling of *U. maydis*^38^ wild-type (Fig. 3, **B3(a)**) and Pcrg1:grx4 (Fig. 3, **B3(b)**) strains grown in minimal medium for 24 h displayed downregulation of TORC1 pathway activation components.

Taken together, we see that in EDF, activation components RAGULATOR, Rag GTPases and Rheb-TSC complex most often respond to changes in fungal growth environments, alongside amino acid sensing proteins Castor and Sestrin, and KICSTOR complex for TORC1 inhibition (Fig. 3). On the other hand, much of the expression in Dikarya is seen for the GATOR/SEA complexes of TORC1 inhibition, with Rag GTPases and Rheb responding for activating TORC1 (Fig. 3).

### Phylogenetics of selected TORC1 pathway components

**Fig. 4:**
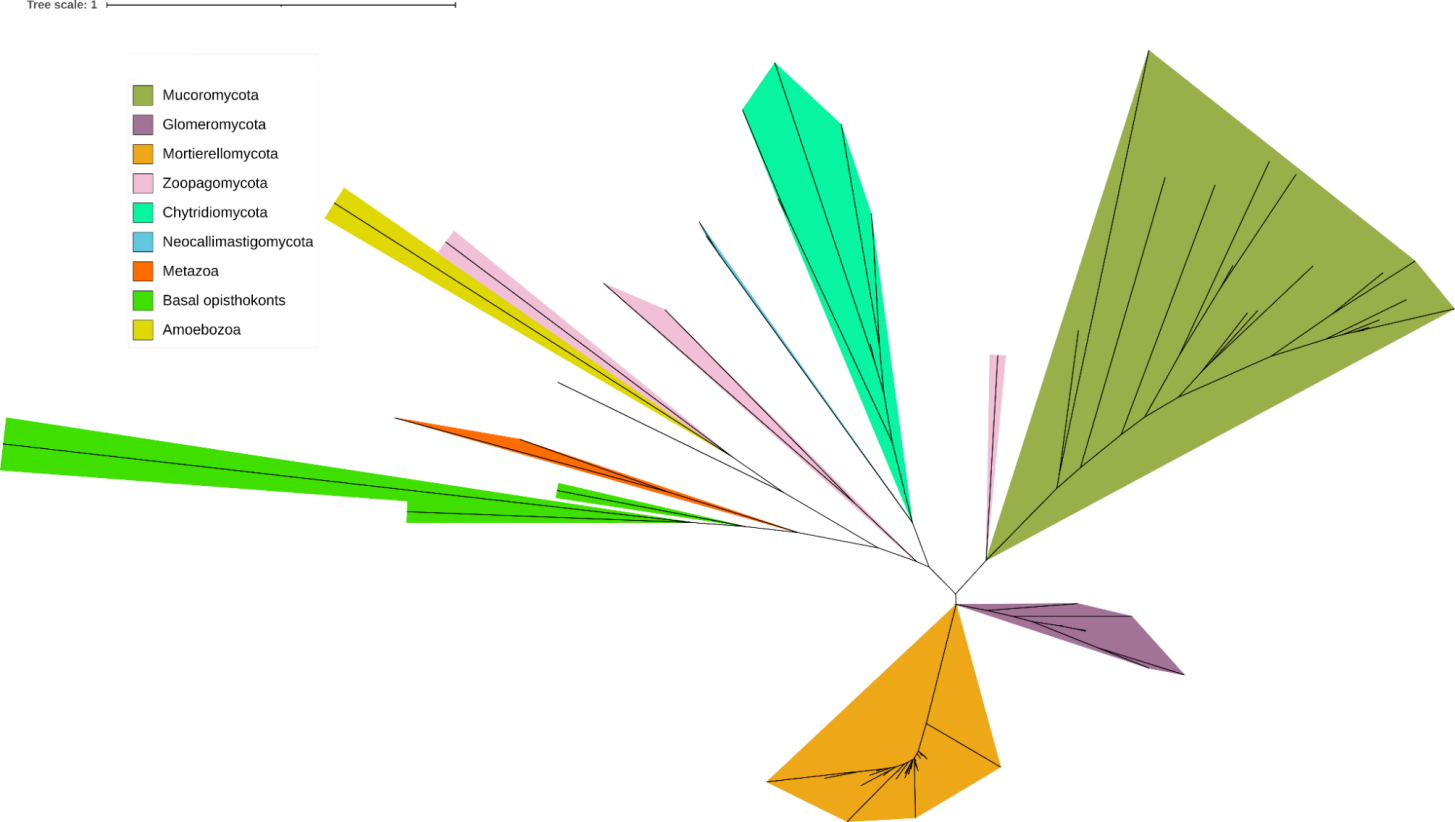
Unrooted maximum likelihood tree for Ltor3 proteins across selected eukaryotic lineages.

In order to understand whether duplications, losses, horizontal gene transfers impacted TORC1 pathway evolution, we performed phylogenetic analyses for all of the studied protein sequence sets (Supplementary file S2). We observed that in general, TORC1 pathway components were inherited vertically. Moreover, most of the TORC1 components occur as single copies and have a conserved domain architecture (an example of the Ltor3 protein phylogeny is shown in Fig. 4) with a few exceptions. For instance, the Rheb gene duplicated at least twice since we found paralogs in EDF and Basidiomycota. The KICSTOR complex protein SZT2 possesses more than two copies in selected Chytridiomycota (*Chytriomyces confervae*; *Rhizoclosmatium globosum*) and all members of Mucoromycota. Latter taxon also showed duplications of Castor2 and Sestrin (Supplementary file S1). The yeast-specific EGO complex proteins were present in most of the Saccharomycotina genera and their phylogenetic trees recapitulated the species phylogeny of the subphylum (Supplementary file S2).

### Speculative TORC1 pathway model in non-dikarya fungi

**Fig. 5:**
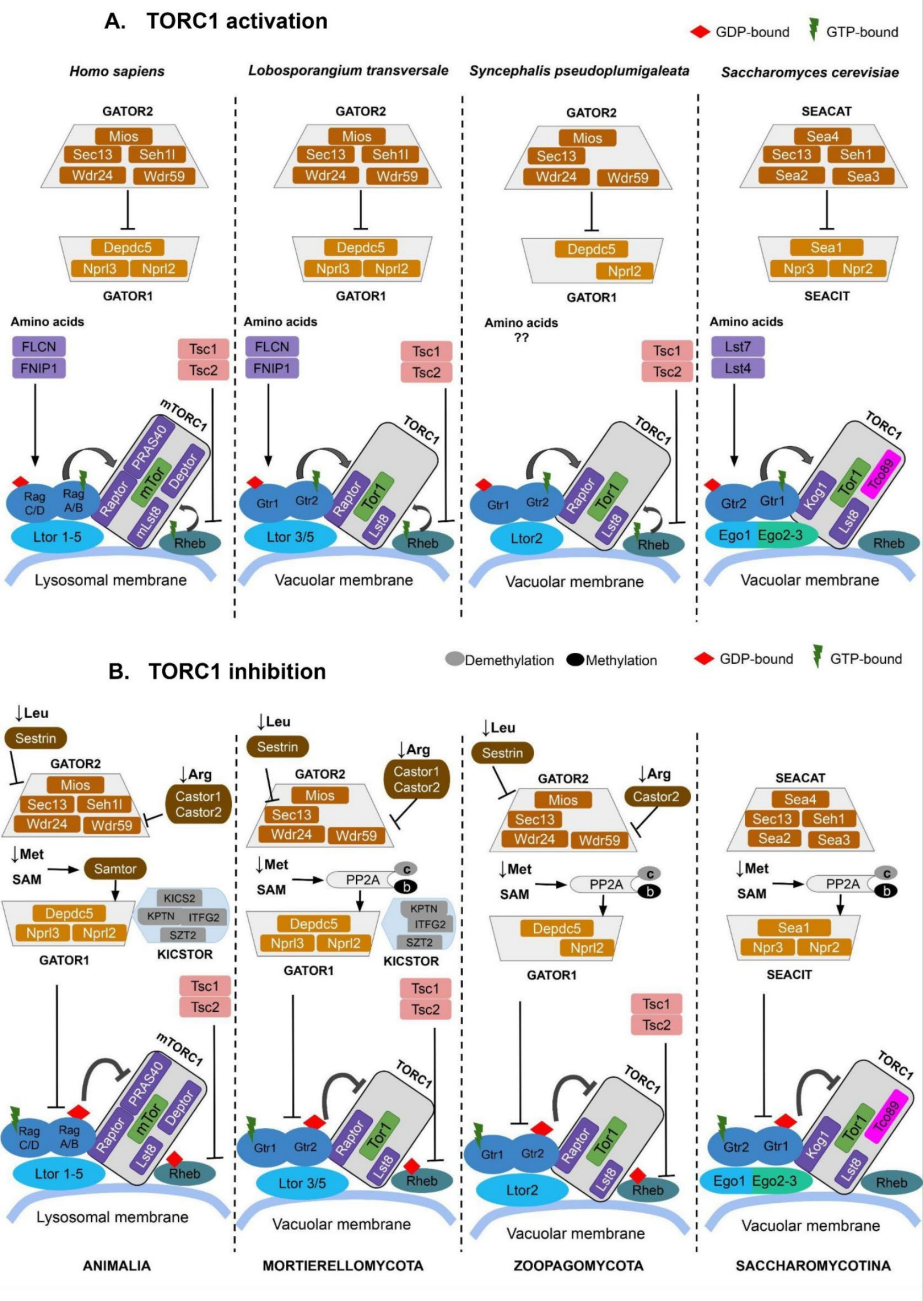
A tentative model of the TORC1 pathway for Mortierello- and Zoopagomycota. Proteins that share homology at the same position are depicted with the same color. The protein nomenclature is in accordance with Table 1.

Taking into account all of the homologs of TORC1 pathway components found in EDF, we propose a model of TORC1 regulation in EDF, taking two phyla as examples (Mortierellomycota: *Lobosporangium transverasale* and Zoopagomycota: *Syncephalis psuedoplumigaleata* respectively, Fig. 5). The presence of upto 2 out of 5 LAMTOR components might facilitate their interaction with Rag GTPases (Gtr1/Gtr2), followed by localization to the vacuolar membrane, thereby promoting TORC1 activation (Fig. 5). The TSC complex would also enable Rheb-TSC mediated TORC1 activation similar to mammalian TORC1. It is unsure how the localization of Rag heterodimers (Gtr1/Gtr2) to the vacuolar membrane and their subsequent binding to TORC1 occurs in the absence of the FLCN/FNIP1 (Lst4/Lst7) complex in Zoopagomycota (Fig. 5). With regards to TORC1 inhibition, the presence of Castor and Sestrin in Mortierellomycota and Sestrin in Zoopagomycota would promote specificity in amino acid sensing like mammalian TORC1 (Fig. 5). The presence of 3-protein KICSTOR instead of the complete 4-protein complex in Mortierellomycota might enable the recruitment of GATOR1 to the vacuolar surface and provide an additional layer of complexity on the TORC1 inhibitory dynamics of this fungal group. Taken together, most lineages possess an intermediate set of TORC1 components distinct from both mammalian and *S. cerevisiae*. Moreover, diverse fungal lineages retained TORC1 subunits typically associated with animals.

## Discussion

The study analyzes the evolutionary dynamics of TORC1 metabolism in the fungal tree of life. We show that EDF not only possesses more TORC1 pathway components than Dikarya, but they also share more similarities with the mammalian TORC1 pathway. The conservation of the ancestral TORC1 pathway varies among fungal lineages, with Mortierellomycota possessing the highest number of mTORC1 pathway components among fungal lineages, followed by Mucoro- and Glomeromycota.

The study by Petit and coworkers revealed that FLCN is specifically required for amino acid stimulated recruitment of mTORC1 to lysosomes by Rag GTPases, while FNIP1 promotes this recruitment and Rag interactions of FLCN^39^. Similarly in the case of *S. cerevisiae*, Lst7/Lst4 complex (FLCN/FNIP1 in humans, Table 1) was involved in trafficking of amino acid permease to the cell surface in response to nitrogen scarcity^40^. The conservation of the FLCN/FNIP1 complex in Mucoro-, Entomophthoro- and Neocallimastigomycota coupled with their upregulation in stress experiments of Mucorales, suggests a potential involvement of this complex in EDF, in line with Saccharomycotina (Fig. 3). The absence of this complex in Basidiomycota puts an open question on whether this phylum evolved an alternative TORC1 activation machinery.

The pentameric LAMTOR/RAGULATOR complex in humans requires the formation of Ltor2-3 and Ltor4-5 roadblock domain dimers, the former assisted by one alpha helix from Ltor1 (Table 1)^9^. The five-protein LAMTOR complex is reduced to four and three protein complexes in *S. pombe*^41^ and *S. cerevisiae*^42^ respectively. In *S. pombe*, Lam1 (Ltor1 in humans) forms a β-strand and wraps around Lam4 (Ltor5 in humans) and Lam2-3 roadblock heterodimer^41^. The Ego2 and Ego3 of *S. cerevisiae* do not dimerize but are held together by Ego1^9^. The occurrence of Ltor2 and Ltor3 in Mucoro-, Neocalli-, Chytridio-, and Zoopagomycota (Fig. 1) coupled with the differential expression of Ltor2 and Ltor3 in stress environments (Mucoromycota, Fig. 3) points to the conservation of Ltor2-3 canonical roadblock heterodimer in EDF. The absence of Ltor4 in fungi, with the exception of a Chytridiomycota representative (*Spizellomyces punctatus* DAOM BR117: KND00403.1), alongside the occurrence of Ltor5 in Mucoro-, Glomero-, and few Chytridiomycota defers the possibility of formation of Ltor4-5 dimer in EDF. Similarly, lack of Ltor1 in EDF (except two species: *Bifiguratus adelaidae* and *Anaeromyces robustus*) points at a loss of N-terminal fold of Ltor1 that completes the complex structure and enables interaction with Rag GTPases. The occurrence and transcription expression of Ltor2, Ltor3 and Ltor5 in EDF points to a reduction in complexity of the RAGULATOR complex in EDF.

The occurrence of Rheb in all fungal groups except Kickxellomycota and Blastocladiomycota displays the conservation of this protein in the TORC1 pathway. The function of Rheb in *S. cerevisiae* was observed to regulate arginine and lysine uptake^43^. This finding supports the absence of arginine and lysine sensor proteins Castor and Sestrin from *Saccharomycotina* and possibly other Dikarya. It also indicates the function of Rheb beyond Rheb-TSC mode of TORC1 activation.

The conservation of both proteins of the TSC complex in Mucoro- and Neocallimastigomycota, along with Ustilago- and Taphrinomycota extends the possibility for the presence of a functional Rheb-TSC mode of TORC1 activation. The downregulation of Rheb and TSC complex proteins against oxidative stress response in *N. crassa* wild-type strain suggests a potential involvement of the Rheb-TSC mediated TORC1 activation in members of Ascomycota possessing both Tsc1 and Tsc2 (Fig. 3). Tsc2 contains the GAP domain responsible for hydrolysis of GTP-bound Rheb into a GDP-bound inactive form during nutrient starvation. Tsc1 is crucial in stabilization of the Tsc2 dimer and formation of a functional heterodimeric complex that localizes in the lysosomal membranes^16^. Hence, it is to be speculated whether the TSC complex is functionally active in the presence of only Tsc2, as observed in Basidio- and Zoopagomycota.

Studies in *Drosophila melanogaster* found that GATOR1 proteins Nprl2 and Nprl3 interact with each other and Depdc5 for lysosomal localization and inhibit TORC1 in response to nitrogen starvation^44^. The GATOR1 equivalent SEACIT proteins in yeast control nitrogen catabolite repression that downregulates proteins utilizing poor nitrogen sources in presence of preferred sources^11^. The GATOR2/SEACAT complex protein Seh1 (Seh1l) forms a dimer with Sea4 (Mios), inhibits activity of the GATOR1/SEACIT complex and prevents TORC1 inhibition^11^. The absence of Seh1 from selected EDF groups (Mucoro-, Kickxello-, Blastocladio- and Neocallimastigomycota) and Basidiomycota leaves an incomplete view on the mechanism of TORC1 inhibition by GATOR1/SEACIT (Fig. 2). The complete absence of the SEACIT/GATOR1 complex and a majority of SEACAT/GATOR2 complexes from Wallemiomycota (Basidiomycota) points to a loss of SEA/GATOR-dependent regulation of TORC1 in this fungal group (Fig. 2).

The conservation of KICSTOR components in Mucoromycota, along with their up- and downregulation in transcriptomic experiments opens the possibility of similarity between EDF and mammalian mode of TORC1 inhibition by KICSTOR (Fig. 3). Previously, the study by Wolfson et al. in 2017 stated that the KICSTOR is conserved only in vertebrates and not in fungi^13^. Our results expand the conservation of this complex in fungi. While KPTN and ITFG2 form a heterodimer, the SZT2 component of KICSTOR plays a central role in interaction with GATOR1. This localizes the KICSTOR complex to the lysosomal surface, and induces the GAP function of GATOR1^45^. It is difficult to speculate whether the absence of one or more KICSTOR components could keep the complex active in fungal phyla Kicxello-, Zoopago-, Blastocladio- and Chytridiomycota.

Structural studies on Castor proteins revealed that Castor1 possesses arginine binding pockets and recognises arginine deprivation. Castor2 shares 63% similarity with Castor1 but lacks arginine recognizing sites^17^. Castor1 forms a homodimer and a heterodimer with Castor2, and together they bind to GATOR2 to induce TORC1 inhibition. The existence of Castor1 in EDF (Mucoro-, Mortierello-, Neocalli- and Entomophthoromycota) along with their up- and downregulation in transcriptomic stress experiments of Mucoromycota provides potential for mTORC1-like amino acid sensing (Fig. 3). However, the presence of only Castor2 in selected EDF and Dikarya does not confirm the extent of arginine sensitivity in fungi (Fig. 2). Structural studies discovered that this mechanism of binding of Castor1 to GATOR2 is similar to the one found in *E. coli* and cyanobacteria^17^. However, Chantranupong and colleagues (2016) found only one Castor-like protein in prokaryotes. This points at a duplication event of an ancestral Castor protein in the common ancestor of animals and fungi, that produced Castor1 and Castor2 in vertebrates and in parallel, loss of Castor1 in selected fungal lineages, including loss of both copies in Dikarya^12^.

Sestrin functions as an intracellular leucine sensor that negatively regulates the TORC1 signaling pathway through the GATOR complex. It is known that Sestrin evolved in the last common ancestor of animals, fungi and amoebozoa and was subsequently lost in the ancestor of *S. cerevisiae*^46^. The yeasts exapted leucyl tRNA synthetase (LeuRS) as the leucine sensor for TORC1 regulation. The conserved repertoire of Sestrin in members of phyla Mucoromycota points to their similarity to leucine sensitivity in mTORC1. While yeasts have adopted LeuRS as the leucine sensor, EDF have the ancestral Sestrin as leucine sensor (Fig. 3).

The yeast-specific novel TORC1 component Tco89 was also found in selected EDF groups (Mucoro- and Blastocladiomycota) (Fig. 2). Tco89 interacts with GTP-bound Gtr1 (RagA/B in humans, Table 1) and helps in maintaining cellular integrity of the cell during nutrient starvation^6^. The upregulation of Tco89 homologue in *Mucor lusitanicus* alongside Tor1 during growth in anaerobic conditions suggests its possible role in non-Dikarya fungi (Fig. 3).

TORC1 pathway is a critical metabolic step involved in regulating cellular life - development, survival, senescence, tumorigenesis, and inflammation. Rapamycin is a natural product with a broad range of biological activities such as immunosuppressive, antitumor, neuroprotective/neuroregenerative, and lifespan extension. It inhibits the TORC1 pathway, influencing the cascade of reactions having major roles in cell survival. Rapamycin exhibits inhibitory activity against opportunistic pathogenic Dikarya (*Cryptococcus neoformans*)^47,48^, and non-Dikarya (*Mucor circinelloides*)^49^. Our study showed that TORC1 genes respond in different ways to infection related conditions in *M. lusitanicus*, *R. delmar* and *R. microsporus* pointing at differentiation of the pathway regulation within Mucorales. Perhaps the background conditions play a subtle role in TORC1 pathway regulation. For instance, in the rice pathogen, *Fusarium fujikuroi*, rapamycin inhibits TOR pathway depending on nitrogen source conditions and concentrations^50^. In our study we found that all of the TORC1 genes are expressed at the same level in *R. solani* AG1-IA transcriptome profiling upon infecting rice leaves (Fig. 3).

The TSC complex is indispensable for pathogenicity in fungi, as reported from studies in *Fusarium oxysporum* highlighting the inappropriate activation of TORC1 upon deletion of Tsc2^51^. The effect of TSC complex deletion was also observed in *Trichoderma atroviride* in the form of reduced mycoparasitic overgrowth and diminished production of secondary metabolites^52^. The TSC complex in EDF and selected Dikarya may add in regulation of the energetics of the cell with a possible role in pathogenicity. However, the role of this regulation is not the same for all infection models and may depend on the experimental conditions.

TORC1 pathway is a key energy regulator in the cell and as such, is of interest for the optimization of biotechnological procedures. It has been proven that in microalgae, triacylglycerols (TAGs) and starch accumulation is induced by TORC1 inactivation^53^. In the fungal kingdom, white-rot fungus *Phanerochaete chrysosporium* (Basidiomycota) was proved to secrete different extracellular proteins (copper radical oxidase and GMC oxidoreductases, glycoside hydrolases as well as amylase) in response to rapamycin TOR pathway inactivation^54^. But the effect of modulating the TORC1 pathway using rapamycin can be even more promising. It was shown that aflatoxin production in *Aspergillus flavus* is inhibited with rapamycin *via* TORC1 inactivation^55^. Information about the composition of TORC1 in different fungal phyla can impact future biotechnological applications. For instance, oil producing fungi such as Mortierello- along with Mucoromycota, may accumulate more oils in response to mTORC1-like inhibition (Fig. 5)^56^. According to our findings biotechnological settings should take into account differences in TORC1 architecture and regulation among individual organisms to achieve optimal bioproduction.

Having a mTORC1-like regulation with additional inhibitory complexes and amino acid sensors gives EDF a higher chance of phenotypic and regulatory plasticity in response to environmental conditions. Zhang et al. (2017) reported that yeast strains with reduced TOR signaling exhibited enhanced mitochondrial ROS during growth phase^57^. Taken together, the complexity of TORC1 regulation in fungi could pave the way for the flexibility of metabolic reprogramming along with balancing energetics and stress resistance (Fig. 5).

## Methods

### Homologous sequence search

The TORC1 pathway reference dataset was created using whole proteomes of model organisms: *Homo sapiens* (GCF_000001405.40), *Mus musculus* (GCF_000001635.27), *Saccharomyces cerevisiae* (GCF_000146045.2) and *Schizosaccharomyces pombe* (GCF_000002945.1). Individual records of the proteins were acquired from UniProt^58^, SGD^59^ and PomBase^60^ databases respectively (Supplementary file S1). They served as BLASTp queries (e-value < = 1e-5) against 183 proteomes of diverse fungi. For each set of proteins, CLANS clustering was performed (p-value threshold of 1e-10, expulsive force exponent set to 2) to remove potential nonspecific hits and group together the fungal homologs. Sequence groups were inspected for protein domain conservation and domain architecture using NCBI’s conserved domain database^61^ and PfamScan assessed by pfam_scan.pl^62^. For proteins displaying sequence divergence (Tco89, EGO complex), protein profiles were created using hmmpress^63^. They were scanned against the proteomes using hmmscan and the true positives were extracted by clustering of the resulting hits in CLANS. The EGO complex homologs were identified in a set of 1154 Saccharomycotina proteomes downloaded from the Y1000 + project^64^. To confirm the loss of genes, we searched the genomes with tBLASTn using proteins from the closest relative as queries.

### Multiple sequence alignment and phylogenetic tree construction

Presence of TORC1 proteins were also searched in five basal Opisthokonts [*Capsaspora owczarzaki* (GCF_000151315.2)^65^, *Sphaeroforma arctica* (GCF_001186125.1)^66^, *Fonticula alba* (GCF_000388065.1)^66^, *Salpingoeca rosetta* (GCF_000188695.1)^67^, *Monosiga brevicollis* (GCF_000002865.3)^68^) as outgroups during phylogenetic tree construction. Multiple sequence alignments were performed for the proteins using MAFFT (v7.407)^69^ local alignment method with a maximum number of iterative refinements set to 100. Phylogenetic trees were constructed with IQ-TREE (v1.6.9)^70^ using maximum likelihood method with automated model selection and ultrafast bootstrap. A phylogenomic species tree was built with OrthoFinder^71^ (with mmseqs) for a set of 39 fungal taxa, 5 basal Opisthokonts, *Amphimedon queenslandica* (GCF_000090795.2), *Homo sapiens* (GCF_000001405.40), *Mus musculus* (GCF_000001635.27), *Caenorhabditis elegans* (GCF_000002985.6), *Drosophila melanogaster* (GCF_000001215.4), *Pomacea canaliculata* (GCF_003073045.1) with *Dictyostelium discoideum* (GCF_000004695.1) as outgroup. The trees were visualized using the iTOL online tool^72^. Lam1 from *S. pombe* was used as an outgroup for Ego1 tree. In the case of Ego2 and Ego3, the trees were rooted with the most ancestral Saccharomycotina representative having each of the proteins.

### Transcriptomic data analysis of TORC1 homologs

The expression of TORC1 pathway proteins was checked in public transcriptomic datasets of EDF and Dikarya. Data was available in the form of transcriptomes from diverse cultural conditions for *Mucor lusitanicus* MS12, *Rhizopus microsporus* FP469, *Rhizopus delemar* 99–880, *Gigaspora rosea* DAOM 194,757, *Rhizophagus irregularis* DAOM 197198, *Trichoderma reesei* QM6a, *Neurospora crassa* OR74A, *Coccidioides immitis* RS, *Orbilia oligospora* ATCC 24,927, *Coprinopsis cinerea* okayama 7-130, *Rhizoctonia solani* and *Ustilago maydis* 521. The data was downloaded as fastq files from ENA server and were quality checked using FASTQC (v0.11.8)^73^. They were adapter-trimmed using fastp (v0.19.6) using default parameters^74^. The fastq reads were then aligned with the reference genome (downloaded from NCBI Datasets) using Hisat2 tool (v2.1.0)^75^. The SAM files obtained from the alignment were compressed into binary file format (BAM) using samtools (v1.10)^76^, and the aligned reads were counted using featureCounts (v1.6.3)^77^. The differential expression analysis was carried out using the DESeq2 R package for protein-coding gene expression from condition-specific transcriptomic datasets^78^. The *Padj* value was set to ≤ 0.05, and RNA-Seq reads were mapped on the protein-coding gene sequences. The log2fold change criteria [*downregulation < 0 > upregulation*] was used to determine the gene expression profiles. The heatmap of the expression profiles was generated using the ggplot2 R package^79^.

## Electronic supplementary material

Below is the link to the electronic supplementary material.

Supplementary Material 1

Supplementary Material 2

## Data Availability

All metadata processed in this study are deposited in zenodo: 10.5281/zenodo.1405166010.5281/zenodo.14051660. All protein identifiers, genomic assemblies, transcriptomic datasets are listed in Supplementary file S1. The figures of phylogenetic trees for TORC1 proteins are depicted in Supplementary file S2.
