# Supplementary material for: Non-dikarya fungi share the TORC1 pathway with animals, not with Saccharomyces cerevisiae
Source: Sci Rep. 2025 Feb 18;15:5926. doi: 10.1038/s41598-025-89635-4 (PMC11836306; doi:10.1038/s41598-025-89635-4)
Supplement: Supplementary file 2 — Supplementary Material 2 [file 41598_2025_89635_MOESM2_ESM.pdf]

Non-Dikarya fungi share the TORC1 pathway with animals, not with *Saccharomyces cerevisiae*

Drishtee Barua, Magdalena Płecha, Anna Muszewska*

Institute of Biochemistry and Biophysics, Polish Academy of Sciences, Pawińskiego 5A, 02-106 Warsaw, Poland

List of images of unrooted maximum likelihood phylogenetic trees inferred with IQTREE2 and rendered with IToL:

FLCN.. 2
FNIP1..2
Ltor2.. 3
Ltor3.. 4
Ltor5.. 5
Rheb... 6
Tsc1.. 7
Tsc2.. 8
KICS2.. 9
ITFG2..10
KPTN.. 11
SZT2... 12
Castor1...13
Castor2...14
Sestrin..15
Tco89..16
Ego1... 17
Ego2... 18
Ego3... 19

FLCN

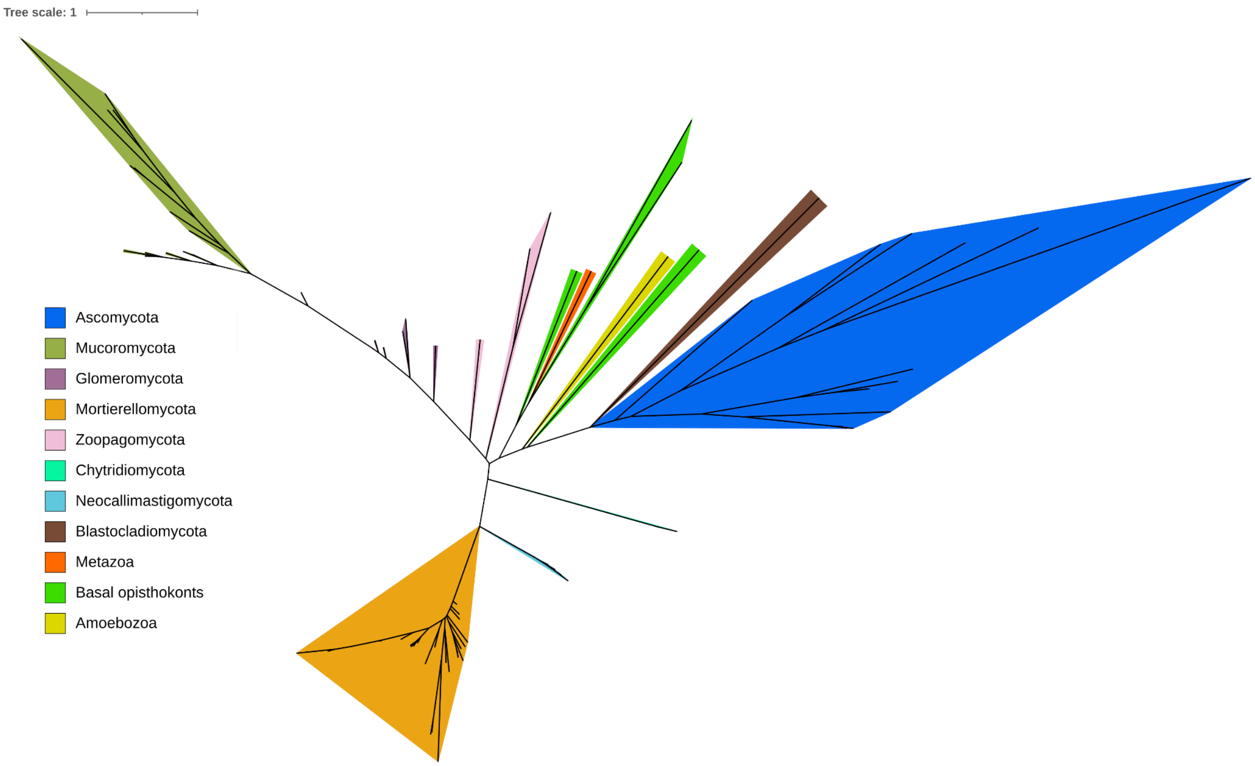
Tree scale: 1
Ascomycota
Mucoromycota
Glomeromycota
Mortierellomycota
Zoopagomycota
Chytridiomycota
Neocallimastigomycota
Blastocladiomycota
Metazoa
Basal opisthokonts
Amoebozoa

FNIP1

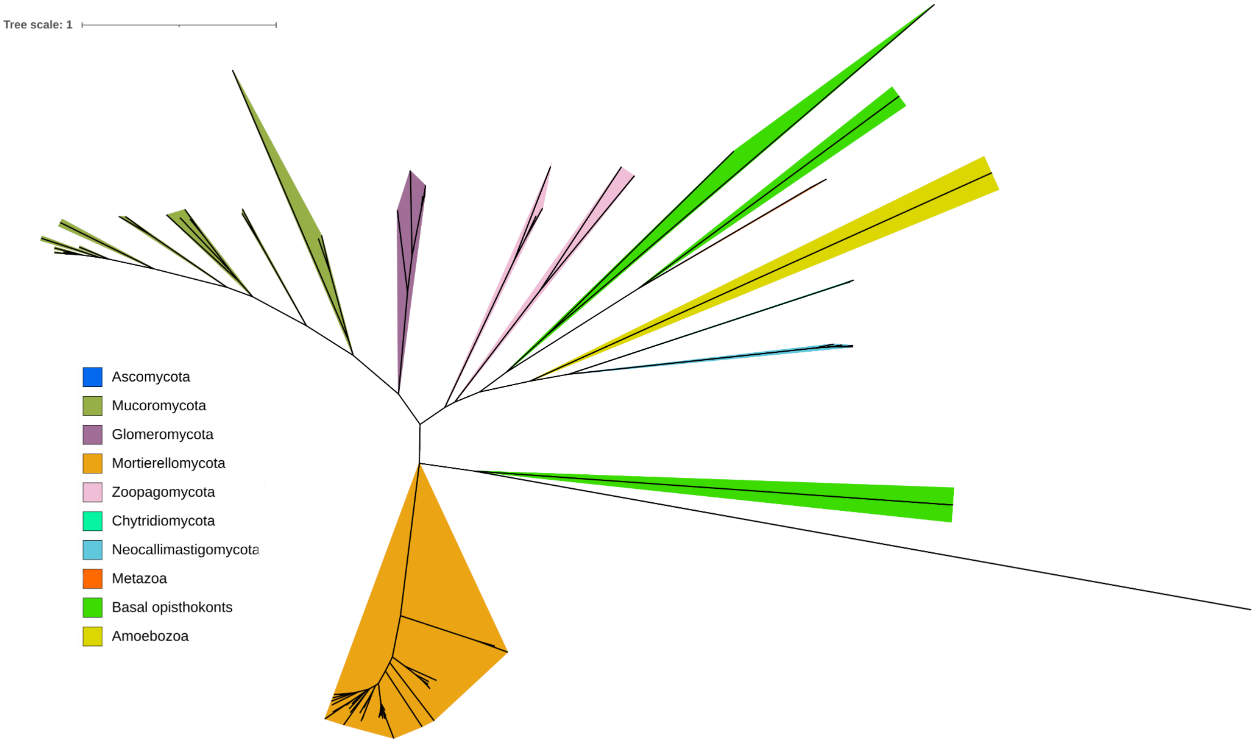
Tree scale: 1
Ascomycota
Mucoromycota
Glomeromycota
Mortierellomycota
Zoopagomycota
Chytridiomycota
Neocallimastigomycota
Metazoa
Basal opisthokonts
Amoebozoa

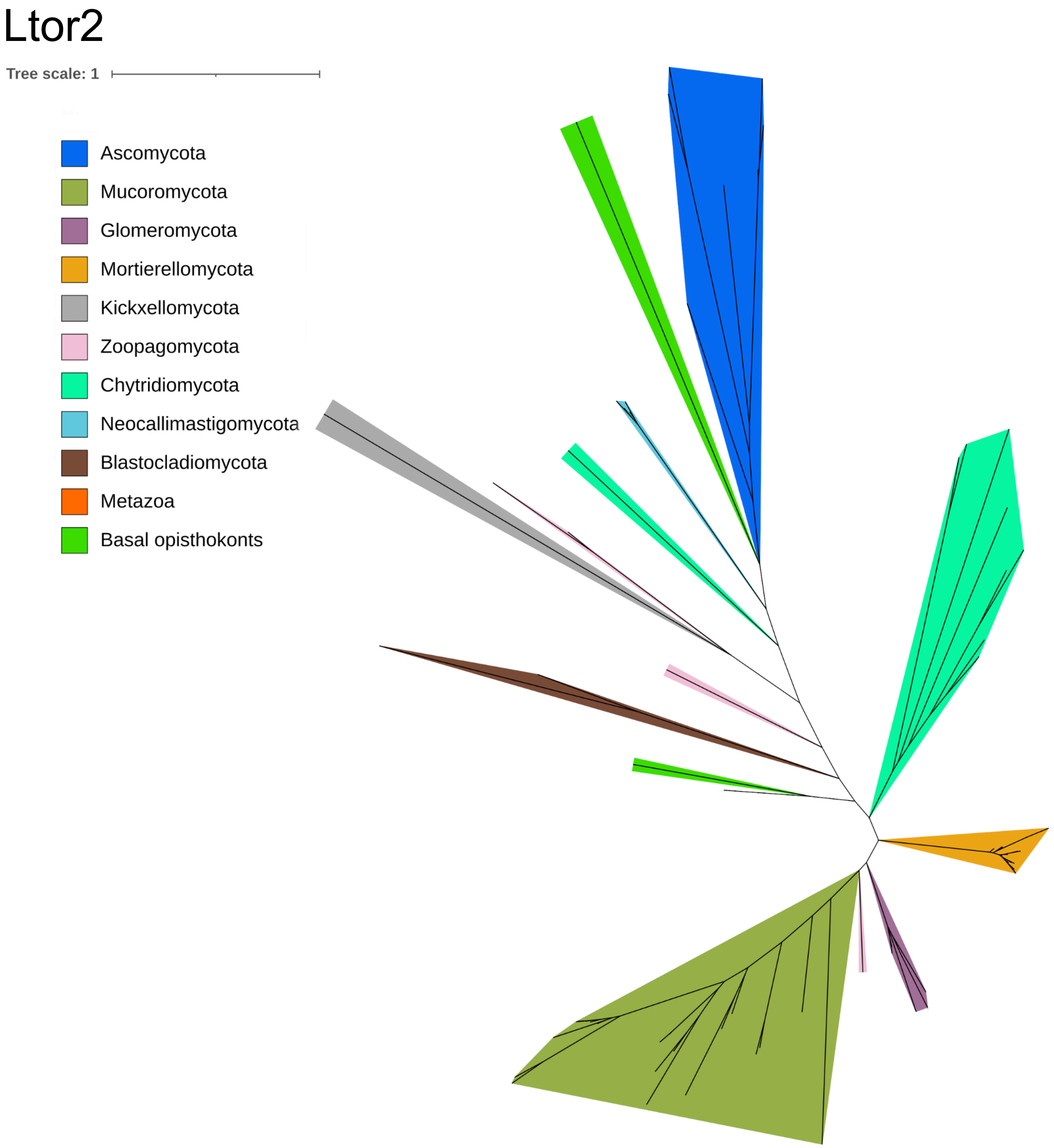
Ltor2
Tree scale: 1
Ascomycota
Mucoromycota
Glomeromycota
Mortierellomycota
Kickxellomycota
Zoopagomycota
Chytridiomycota
Neocallimastigomycota
Blastocladiomycota
Metazoa
Basal opisthokonts

Ltor3

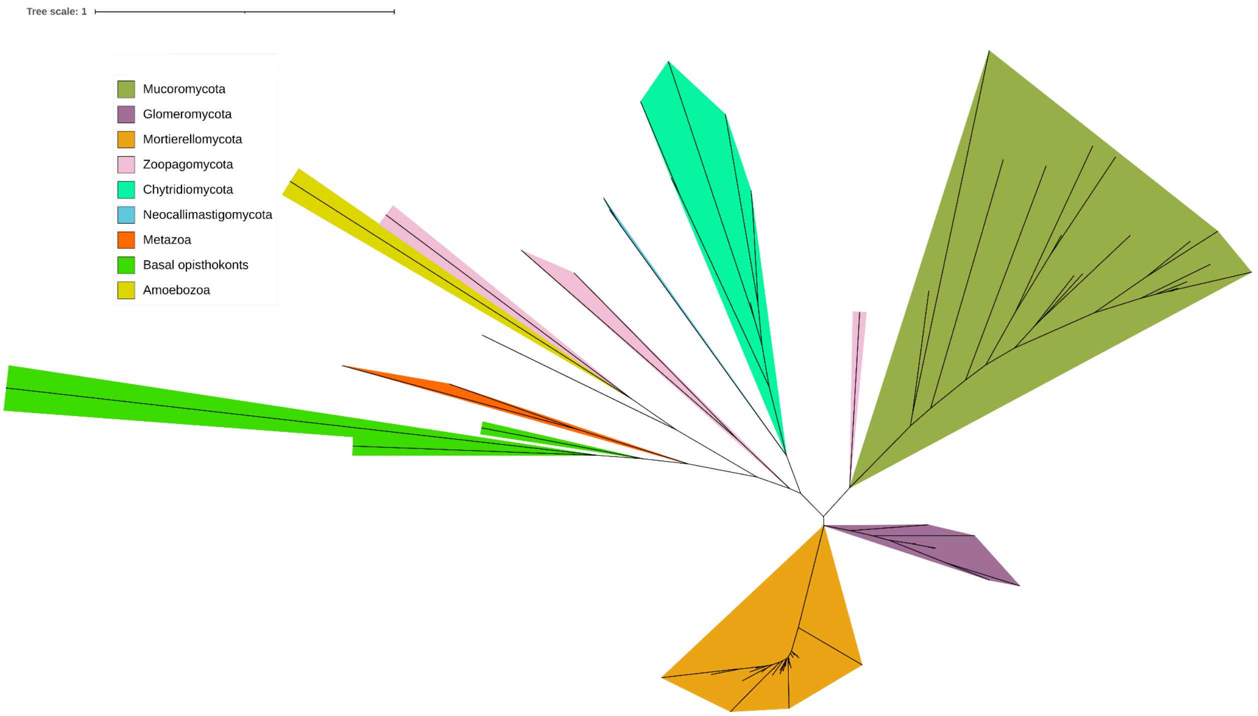

Tree scale: 1
Mucoromycota
Glomeromycota
Mortierellomycota
Zoopagomycota
Chytridiomycota
Neocallimastigomycota
Metazoa
Basal opisthokonts
Amoebozoa

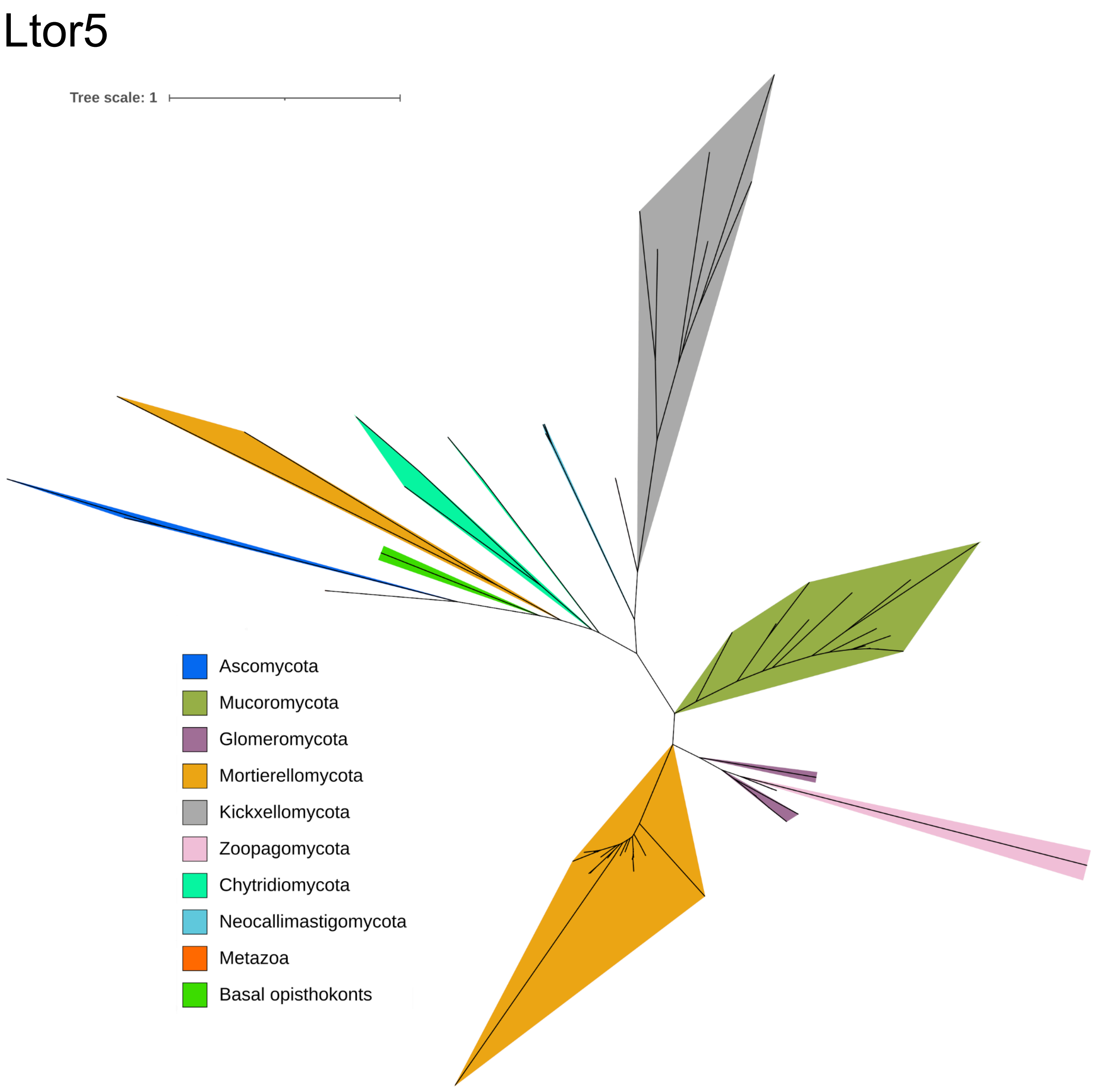
Ltor5
Tree scale: 1
Ascomycota
Mucoromycota
Glomeromycota
Mortierellomycota
Kickxellomycota
Zoopagomycota
Chytridiomycota
Neocallimastigomycota
Metazoa
Basal opisthokonts

Rheb

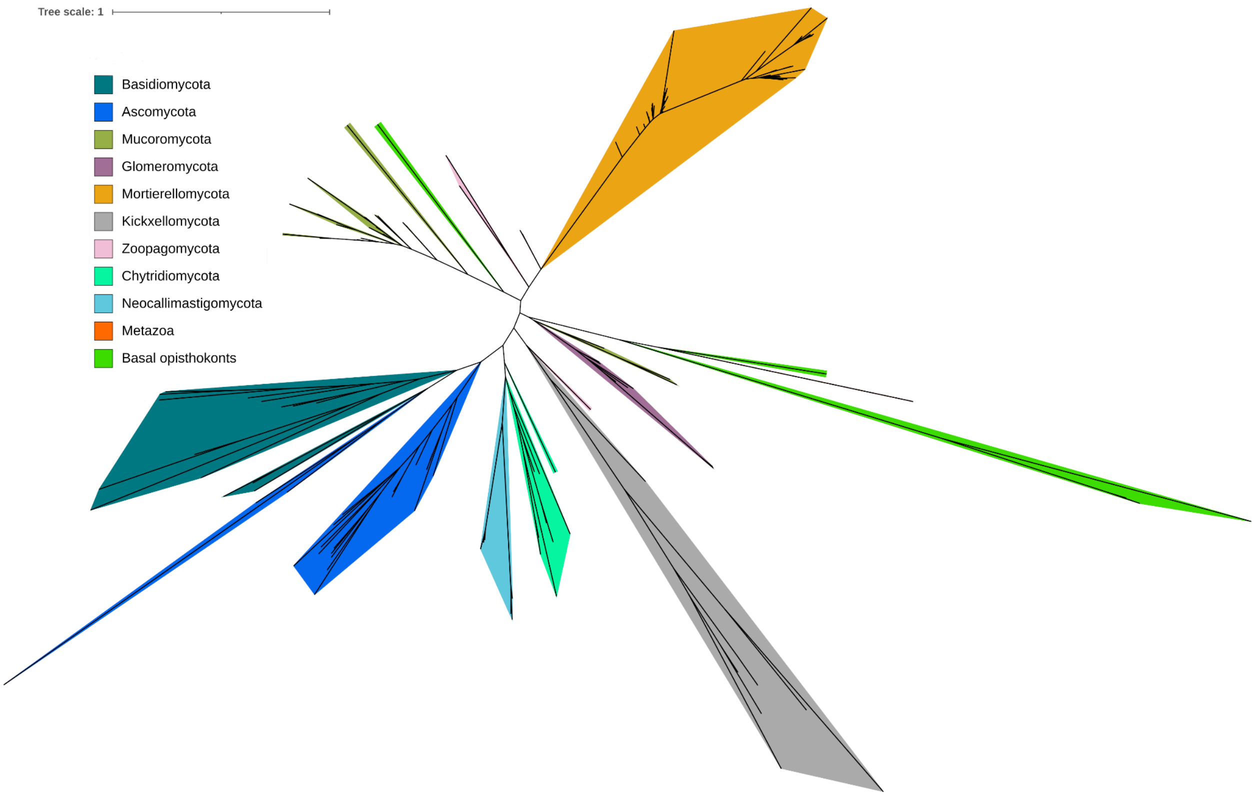

Tree scale: 1
Basidiomycota
Ascomycota
Mucoromycota
Glomeromycota
Mortierellomycota
Kickxellomycota
Zoopagomycota
Chytridiomycota
Neocallimastigomycota
Metazoa
Basal opisthokonts

Tsc1

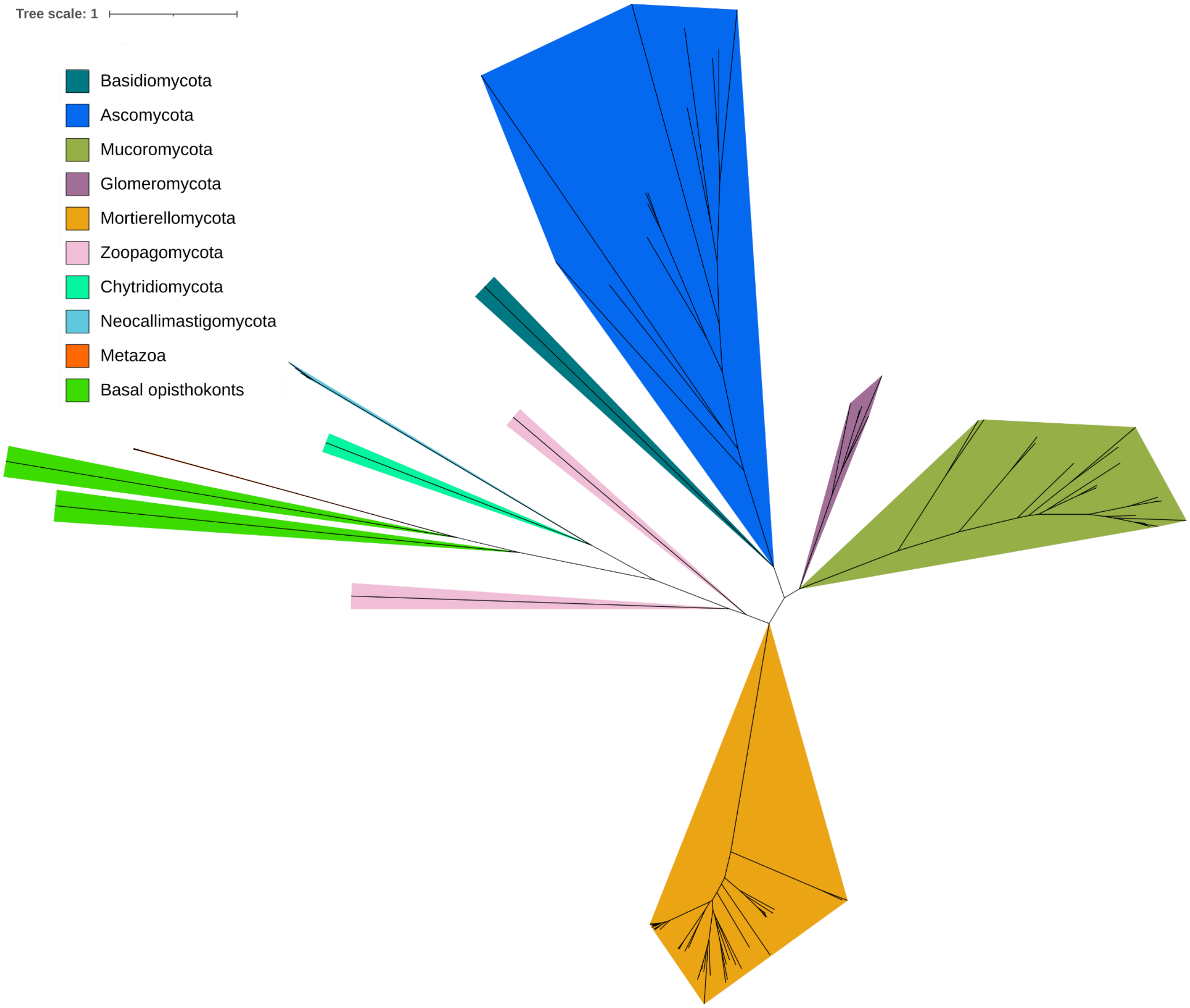
Tree scale: 1
Basidiomycota
Ascomycota
Mucoromycota
Glomeromycota
Mortierellomycota
Zoopagomycota
Chytridiomycota
Neocallimastigomycota
Metazoa
Basal opisthokonts

Tsc2

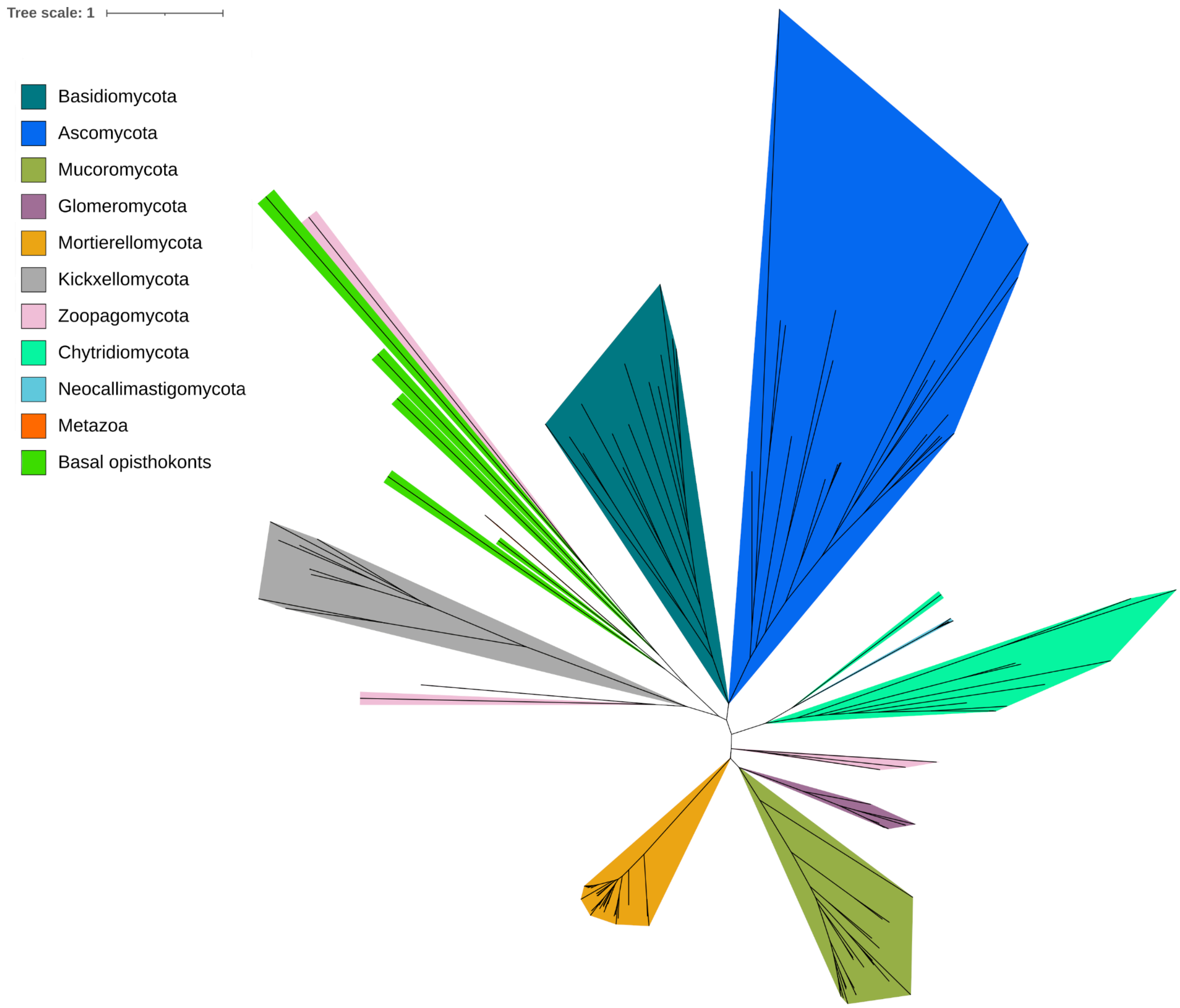
Tree scale: 1
Basidiomycota
Ascomycota
Mucoromycota
Glomeromycota
Mortierellomycota
Kickxellomycota
Zoopagomycota
Chytridiomycota
Neocallimastigomycota
Metazoa
Basal opisthokonts

KICS2

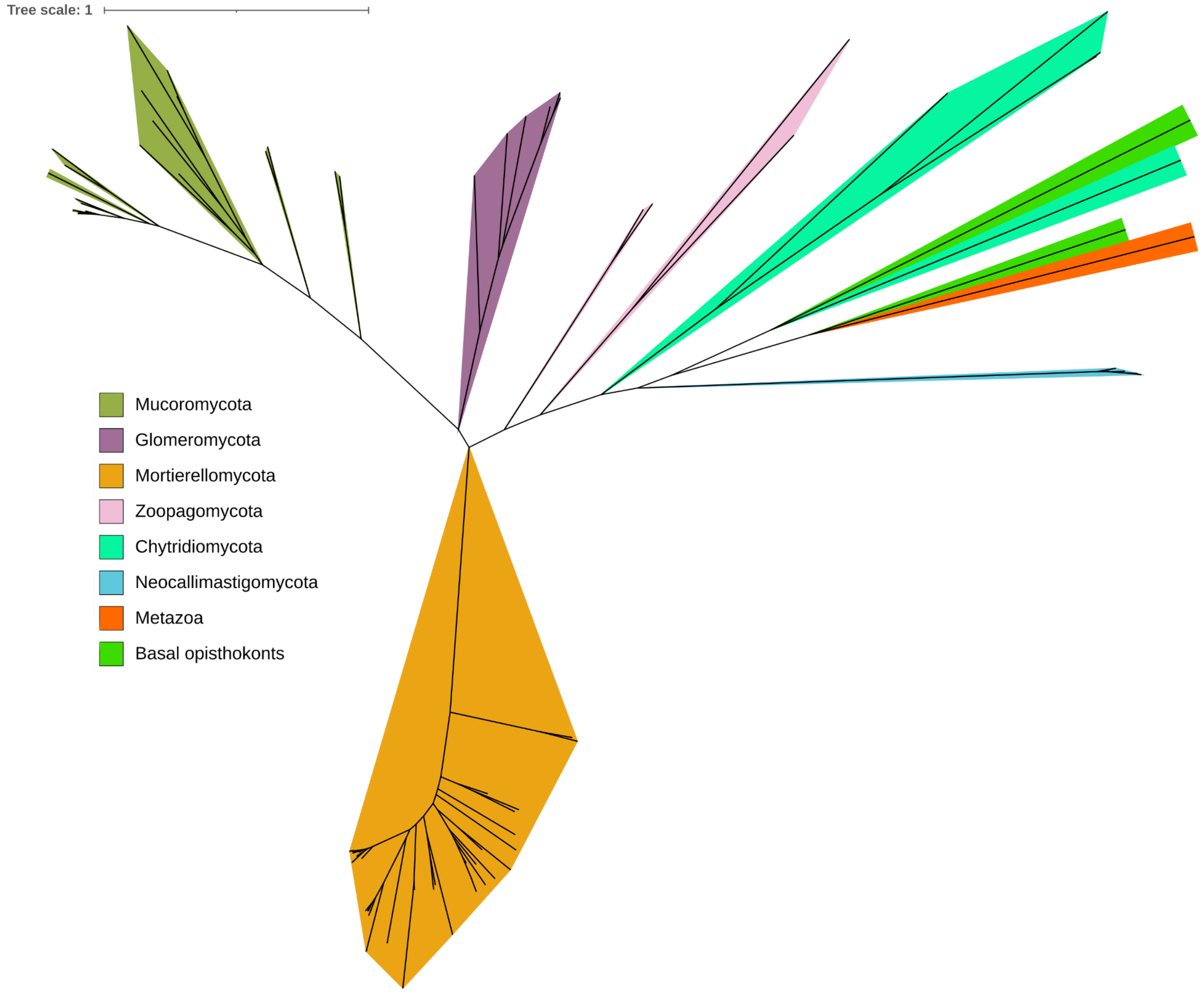
Tree scale: 1
Mucoromycota
Glomeromycota
Mortierellomycota
Zoopagomycota
Chytridiomycota
Neocallimastigomycota
Metazoa
Basal opisthokonts

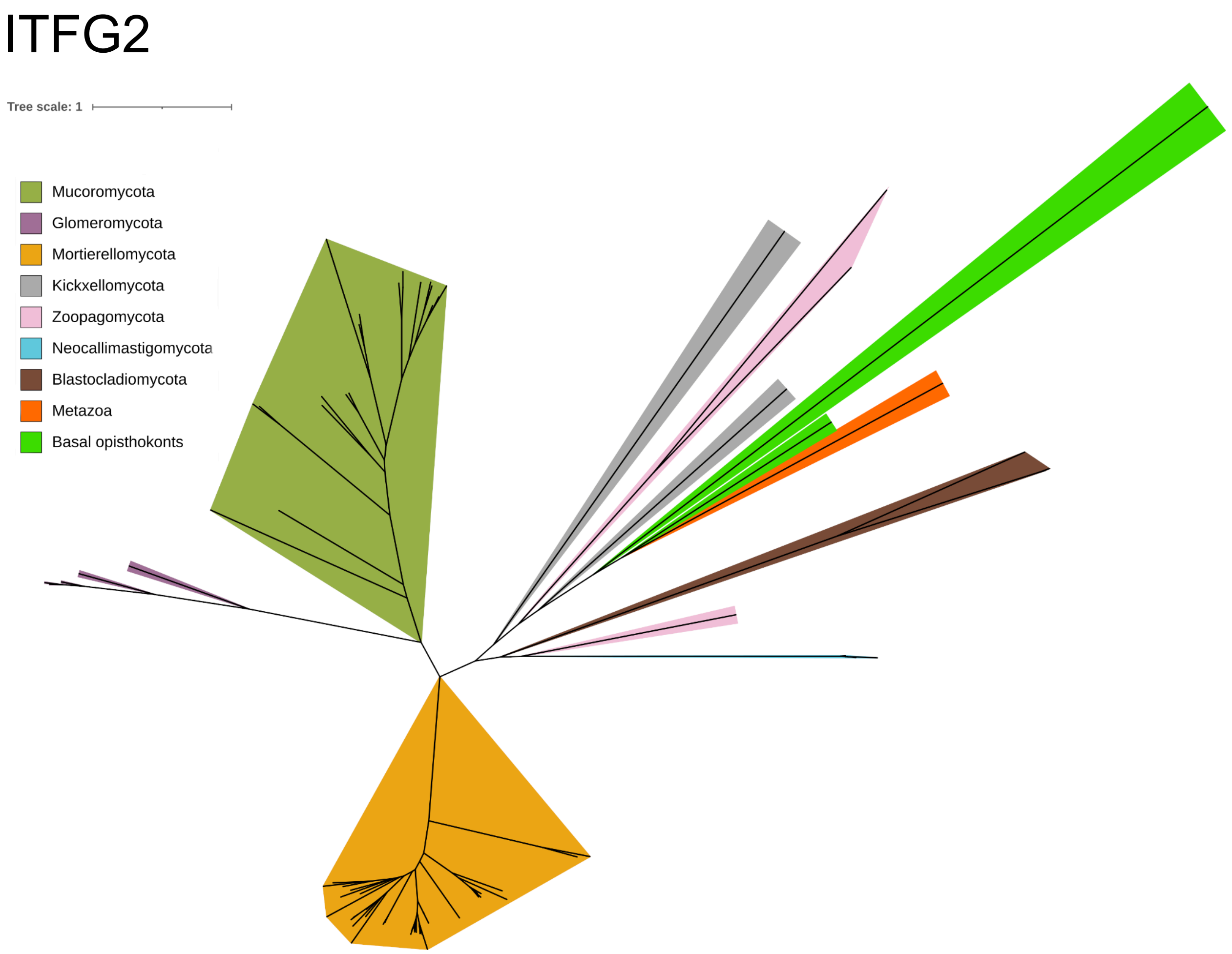
ITFG2
Tree scale: 1
Mucoromycota
Glomeromycota
Mortierellomycota
Kickxellomycota
Zoopagomycota
Neocallimastigomycota
Blastocladiomycota
Metazoa
Basal opisthokonts

KPTN

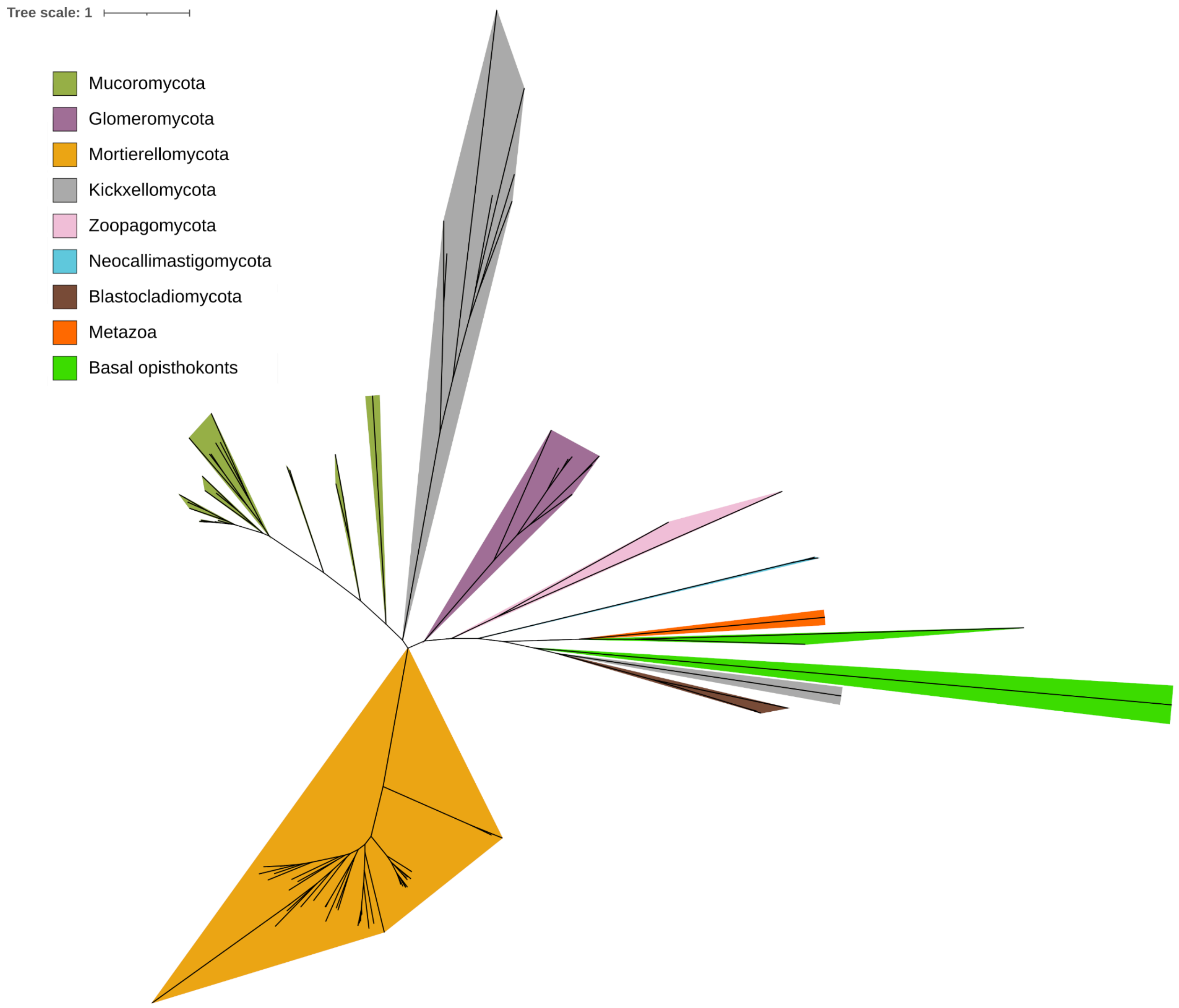

Tree scale: 1
Mucoromycota
Glomeromycota
Mortierellomycota
Kickxellomycota
Zoopagomycota
Neocallimastigomycota
Blastocladiomycota
Metazoa
Basal opisthokonts

SZT2

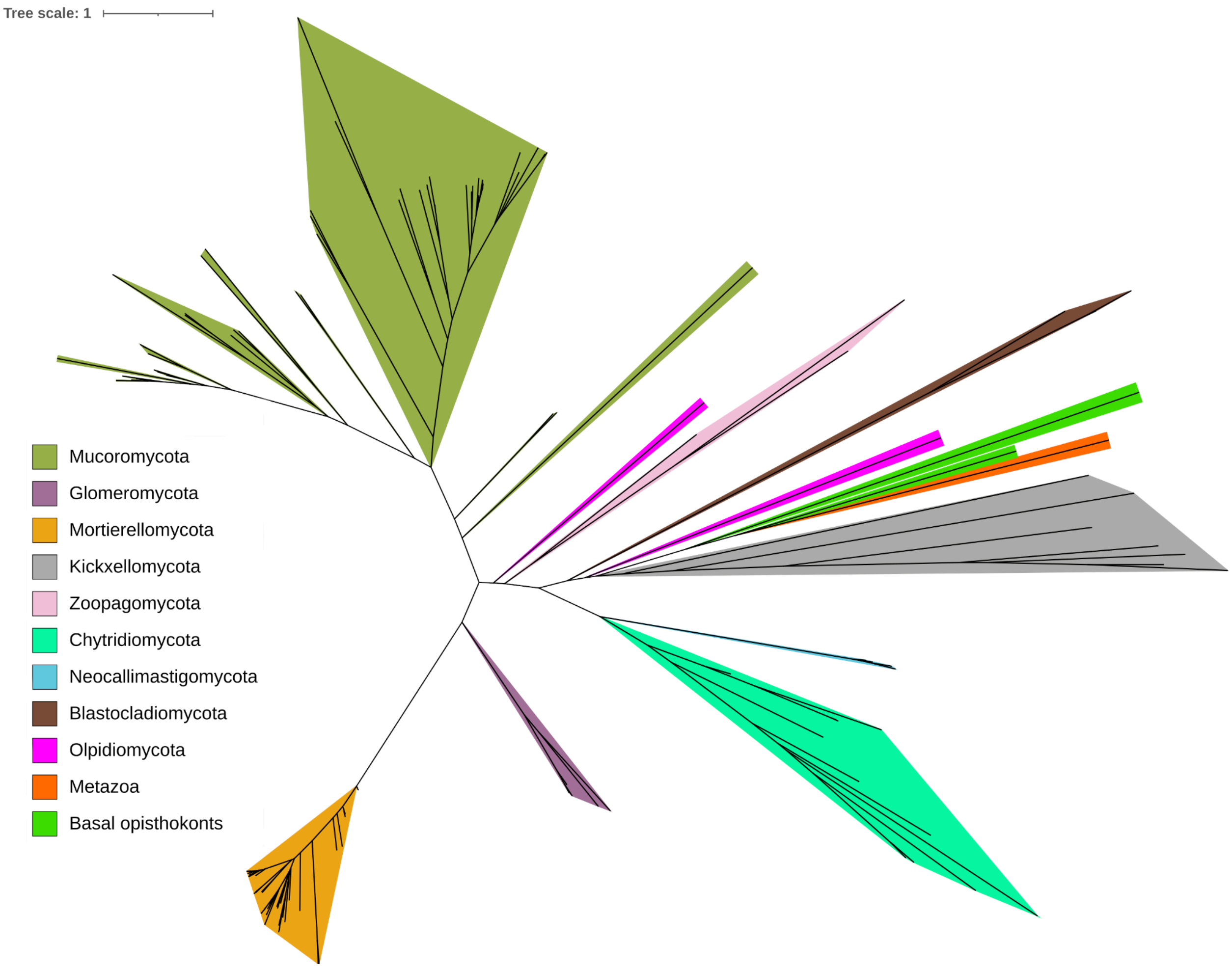

Tree scale: 1
Mucoromycota
Glomeromycota
Mortierellomycota
Kickxellomycota
Zoopagomycota
Chytridiomycota
Neocallimastigomycota
Blastocladiomycota
Olpidiomycota
Metazoa
Basal opisthokonts

Castor1

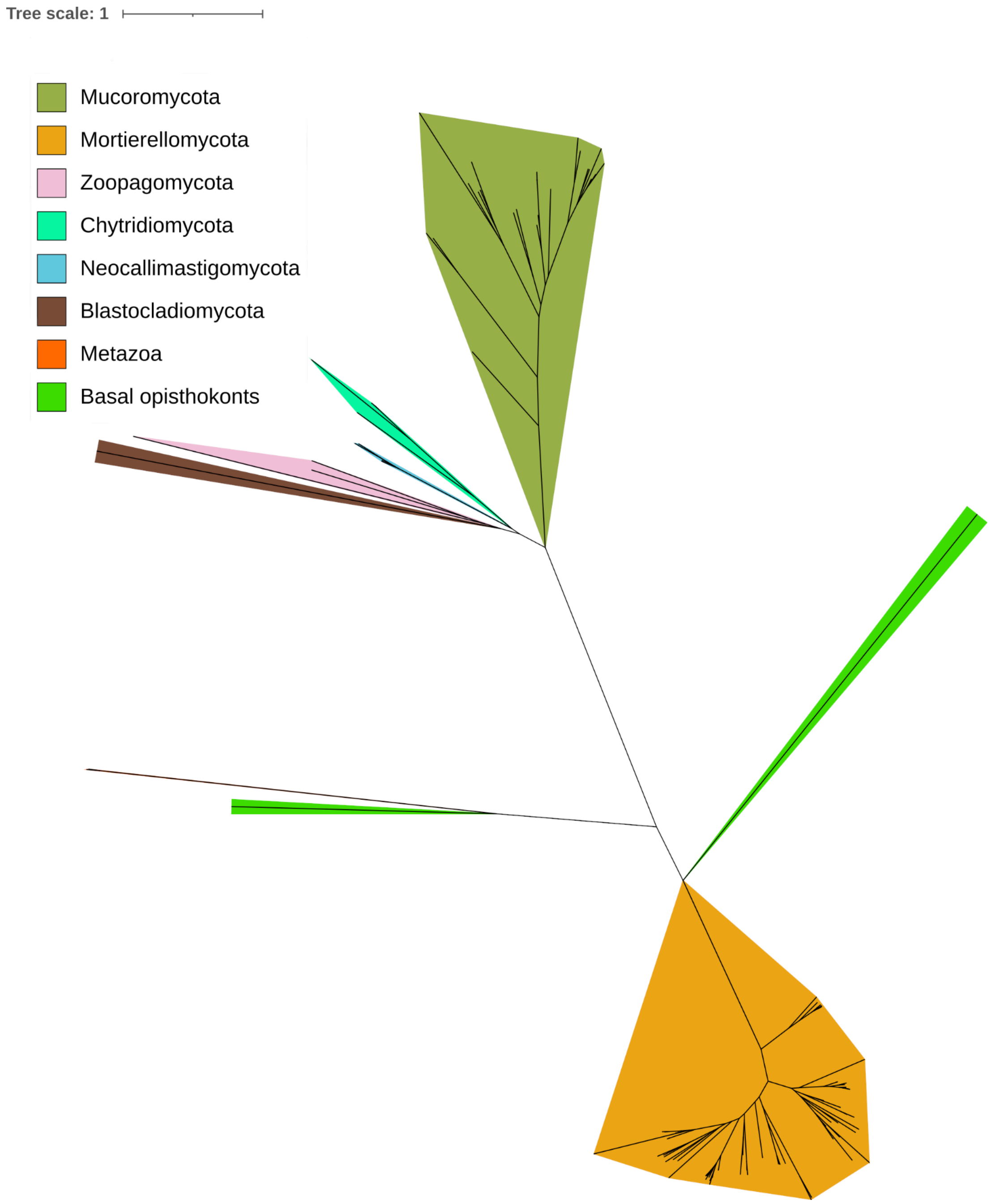

Tree scale: 1
Mucoromycota
Mortierellomycota
Zoopagomycota
Chytridiomycota
Neocallimastigomycota
Blastocladiomycota
Metazoa
Basal opisthokonts

Castor2

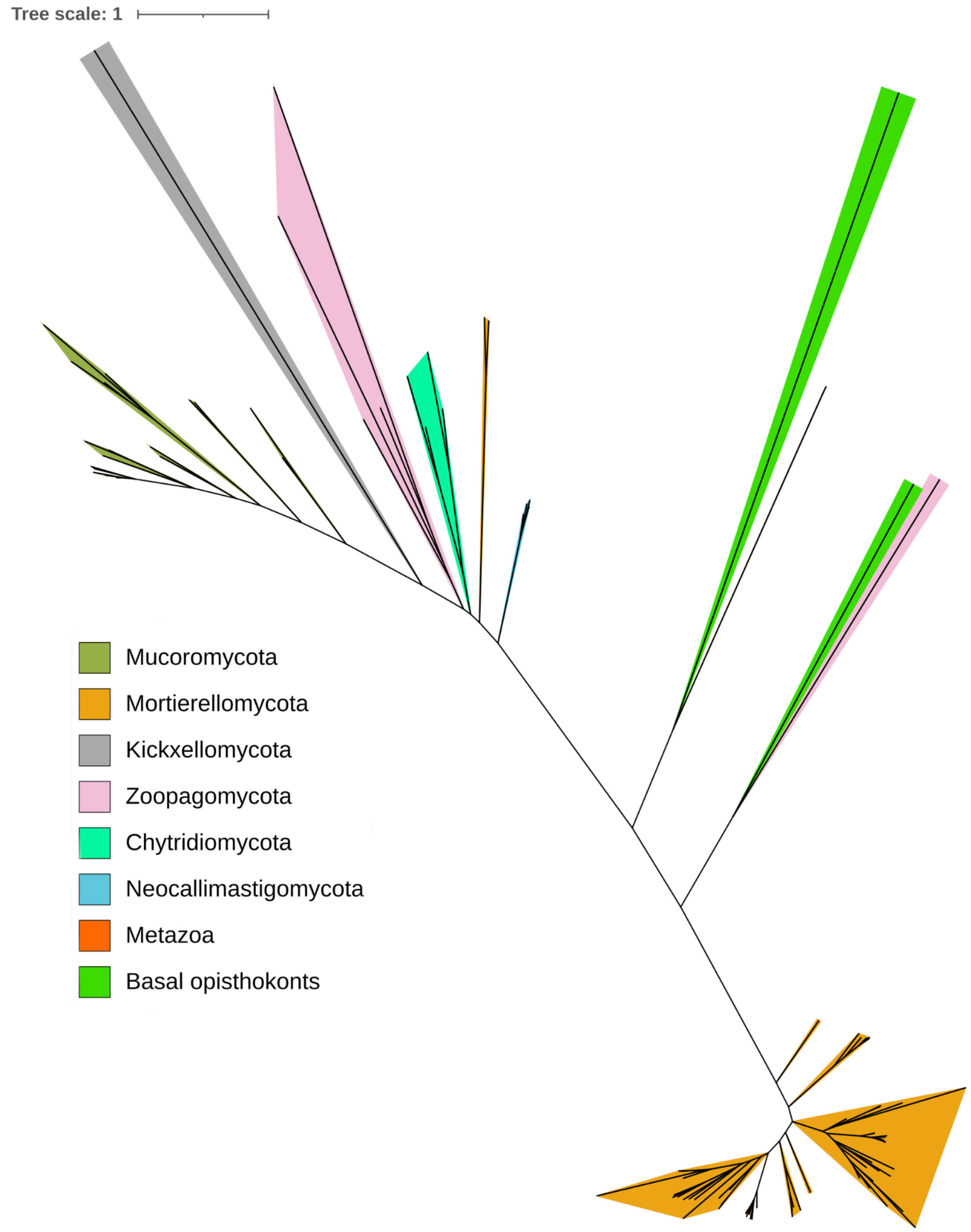
Tree scale: 1
Mucoromycota
Mortierellomycota
Kickxellomycota
Zoopagomycota
Chytridiomycota
Neocallimastigomycota
Metazoa
Basal opisthokonts

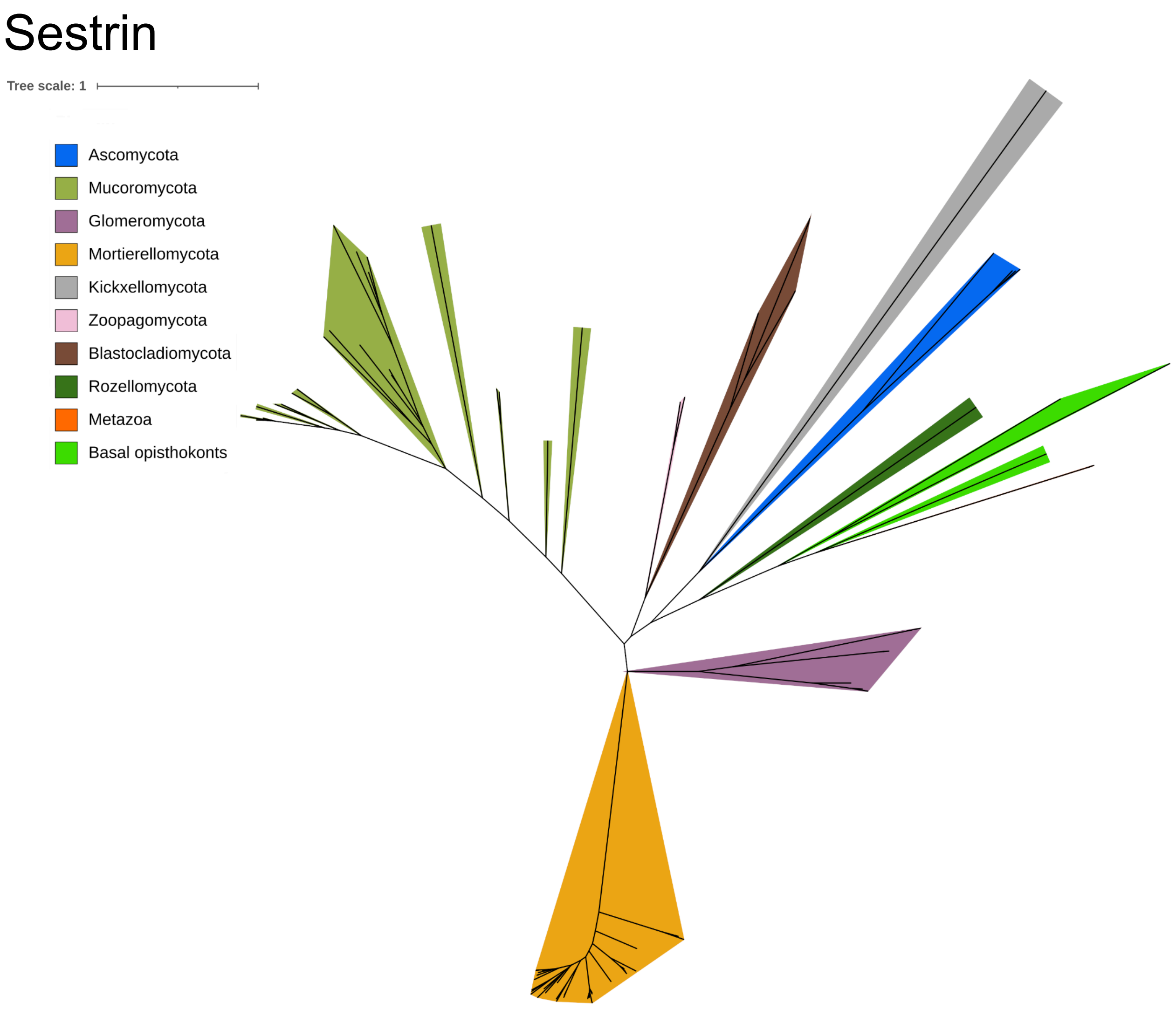
Sestrin
Tree scale: 1
Ascomycota
Mucoromycota
Glomeromycota
Mortierellomycota
Kickxellomycota
Zoopagomycota
Blastocladiomycota
Rozellomycota
Metazoa
Basal opisthokonts

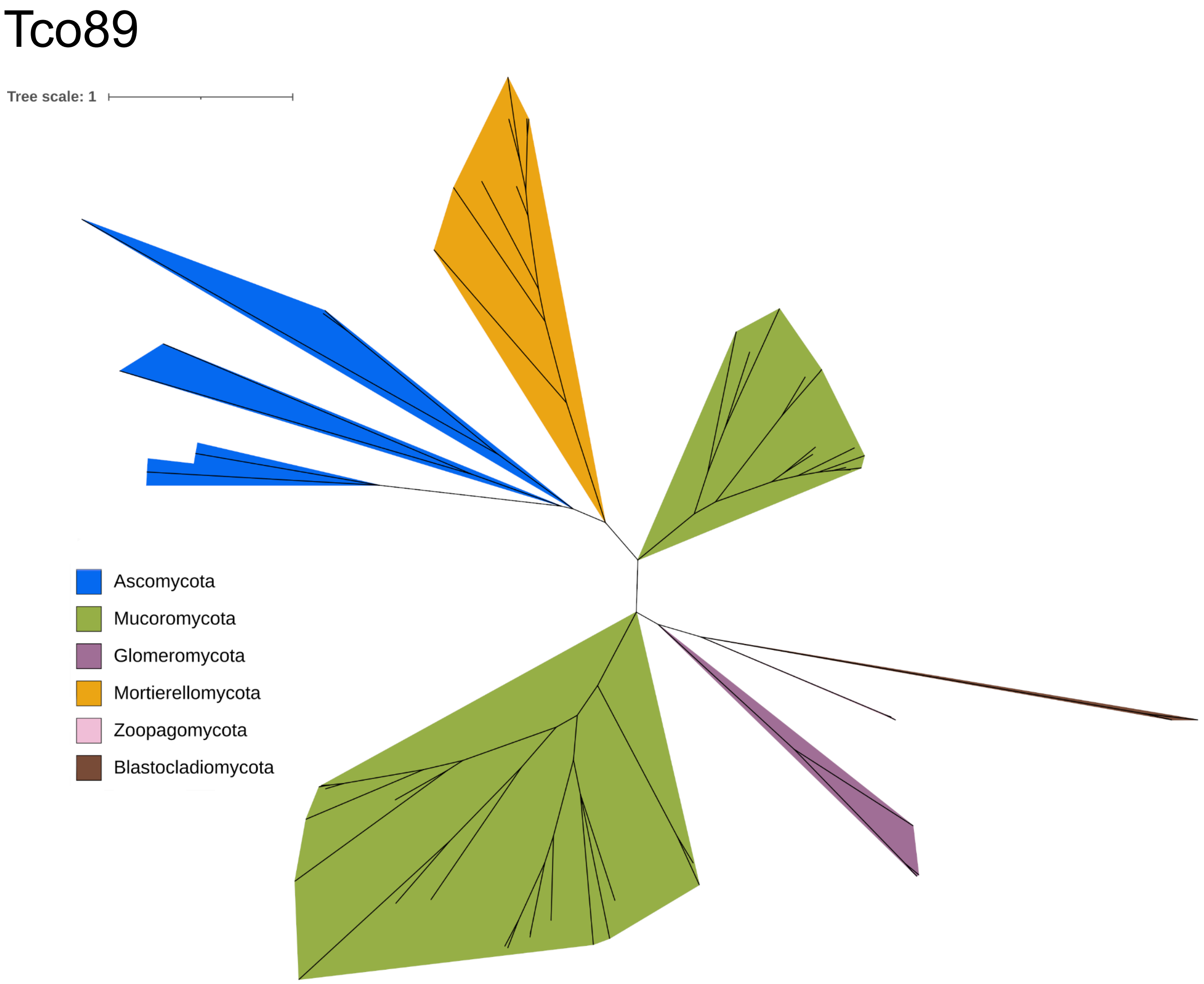
Tco89
Tree scale: 1
Ascomycota
Mucoromycota
Glomeromycota
Mortierellomycota
Zoopagomycota
Blastocladiomycota

Ego1

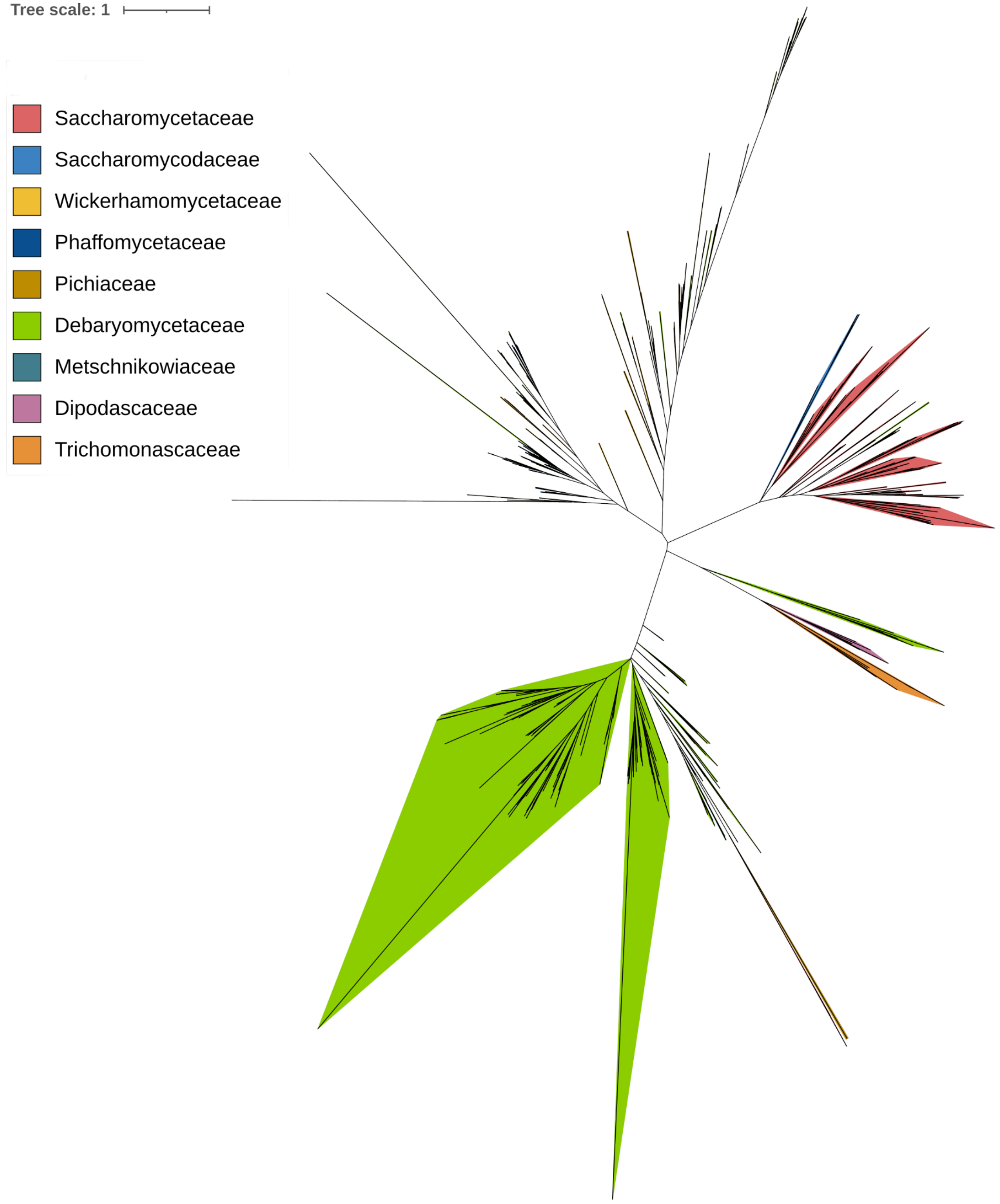

Tree scale: 1
Saccharomycetaceae
Saccharomycodaceae
Wickerhamomycetaceae
Phaffomycetaceae
Pichiaceae
Debaryomycetaceae
Metschnikowiaceae
Dipodascaceae
Trichomonascaceae

Ego2

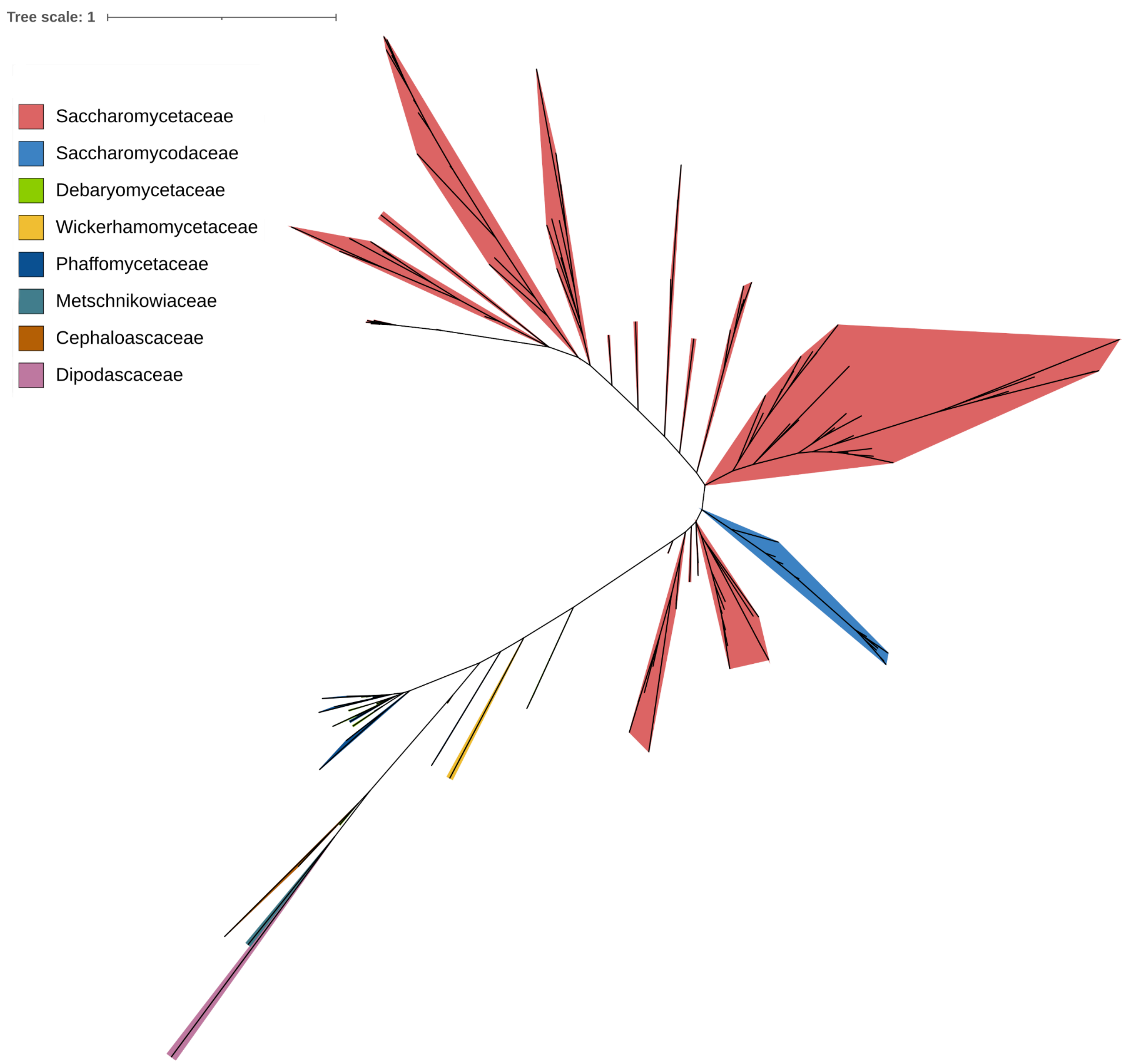
Tree scale: 1
Saccharomycetaceae
Saccharomycodaceae
Debaryomycetaceae
Wickerhamomycetaceae
Phaffomycetaceae
Metschnikowiaceae
Cephaloascaceae
Dipodascaceae

Ego3

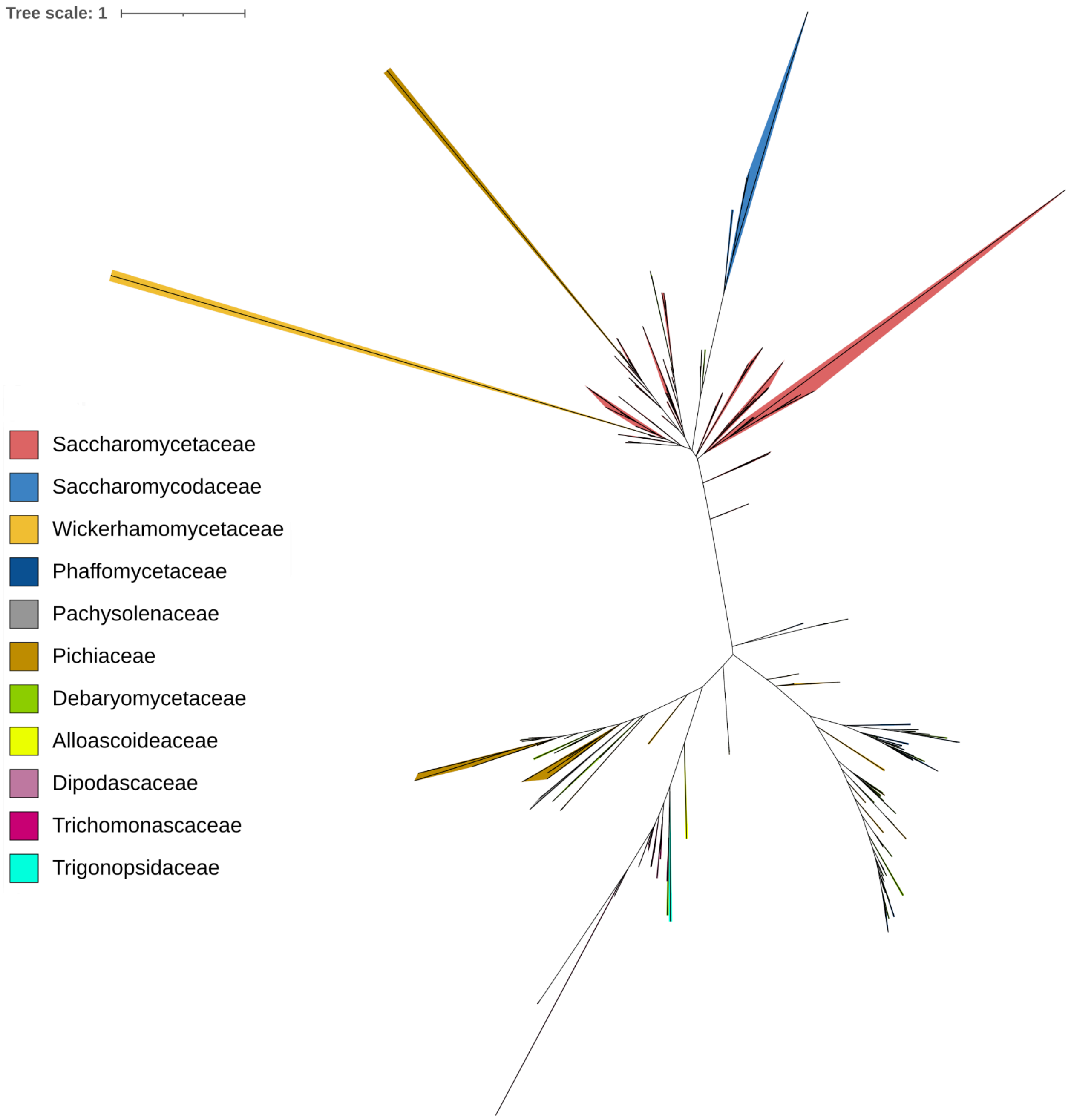

Tree scale: 1
Saccharomycetaceae
Saccharomycodaceae
Wickerhamomycetaceae
Phaffomycetaceae
Pachysolenaceae
Pichiaceae
Debaryomycetaceae
Alloascoideaceae
Dipodascaceae
Trichomonascaceae
Trigonopsidaceae
